# Influenza epidemiology and influenza vaccine effectiveness during the 2014–2015 season: annual report from the Global Influenza Hospital Surveillance Network

**DOI:** 10.1186/s12889-016-3378-1

**Published:** 2016-08-22

**Authors:** Joan Puig-Barberà, Elena Burtseva, Hongjie Yu, Benjamin J. Cowling, Selim Badur, Jan Kyncl, Anna Sominina, on behalf of the GIHSN, Olga Afanasieva, Veronica Afanasieva, Meral Akcay Ciblak, F. Aktas, Selim Badur, Ángel Belenguer-Varea, S. Borekci, Fernando Boza, Elena Bursteva, Zhanna Buzitskaya, Braulia Caetano, Jian Cai, B. Çakir, Mario Carballido-Fernández, Empar Carbonell-Franco, Concha Carratalá-Munuera, S. Çelebi, C. Chai, Ivamara Changas de Lima Porto de Paula, Enfu Chen, Ben Cowling, Yunjie Cui, D. B. Deniz, Elena Dondurei, H. Dong, X. Dong, Mine Durusu, Clotilde El Guerche-Séblain, Fernanda Enda-Moura, A. Eren-Şensoy, Artem Fadeev, Luzhao Feng, Shuo Feng, Raimundo César, Patricia Fisch, Ekaterina Garina, S. Gencer, Vicente Gil-Guillén, Alexa Go, Vitaly Gonchar, Ekaterina Golovacheva, Mikhail Grudinin, M. Hacımustafaoğlu, S. Hancerli, Martina Havlickova, Kristyna Herrmannova, L. Huang, Hui Jiang, Dennis Ip, Helena Jirincova, Lucie Jurzykowska, Lidiya Kisteneva, Ludmila Kolobukhina, Andrey Komissarov, Radka Kralova, Kirill Krasnoslobotsev, Jan Kyncl, Xavier Labrador, Chao Li, Xiangxin Li, Ramón Limón-Ramírez, Jianhua Liu, Mari Carmen, Cédric Mahé, Zdenka Mandakova, L. Merkulova, Sevim Mese, Ainara Mira Iglesias, Evgenia Mukasheva, Angels Natividad Sancho, Lucia Nováková, Elena Obraztsova, Ludmila Osidak, Maria del Carmen, S. Özer, L. Ozisik, Valentina Picot, Maria Pisarev, Florence Pradel, Jitka Prochazkova, Joan Puig-Barberá, Ying Qin, Sonia Raboni, Hana Roháčová, Elena Rozhkova, Sa Li, M.I. Sadikhova, Germán Schwarz-Chavarri, Marilda Siqueira, Elizaveta Smorodintseva, Anna Sominina, Kirill Stolyarov, Vera Sukhovetskaya, G. Sun, Y. Tang, Miguel Tortajada-Girbés, Svetlana Trushakova, José Tuells, Tatiana Tumina, R. Vartanyan, Lubov Voloshuk, Quanyi Wang, D. Wen, Peng Wu, Wen Xiao, Peng Yang, Marina Yanina, Bo Yi, Hongjie Yu, Kubra Yurtcu, Pavel Zarishnyuk, S. Zhang, Yi Zhang, Tiebiano Zhang, Jiandong Zheng, Zhibin Peng

**Affiliations:** 1 Foundation for the Promotion of Health and Biomedical Research in the Valencia Region FISABIO – Public Health, Avda Catalunya 21, 46020 Valencia, Spain; 2 D.I. Ivanovsky Institute of Virology FGBC “N.F. Gamaleya FRCEM” Ministry of Health of Russian Federation, Moscow, Russian Federation Russia; 3 Division of Infectious Disease, Key Laboratory of Surveillance and Early-Warning on Infectious Disease, Chinese Center for Disease Control and Prevention, Beijing, China; 4 School of Public Health, Li Ka Shing Faculty of Medicine, The University of Hong Kong, Hong Kong, Special Administrative Region China; 5 National Influenza Reference Laboratory, Istanbul Faculty of Medicine, Istanbul University, Istanbul, Turkey; 6 National Institute of Public Health (NIPH), Prague, Czech Republic; 7 Research Institute of Influenza, Saint Petersburg, Russian Federation Russia

**Keywords:** Influenza, Virus, Surveillance, Vaccine, Hospitalization, Epidemiological study

## Abstract

The Global Influenza Hospital Surveillance Network (GIHSN) has established a prospective, active surveillance, hospital-based epidemiological study to collect epidemiological and virological data for the Northern and Southern Hemispheres over several consecutive seasons. It focuses exclusively on severe cases of influenza requiring hospitalization. A standard protocol is shared between sites allowing comparison and pooling of results. During the 2014–2015 influenza season, the GIHSN included seven coordinating sites from six countries (St. Petersburg and Moscow, Russian Federation; Prague, Czech Republic; Istanbul, Turkey; Beijing, China; Valencia, Spain; and Rio de Janeiro, Brazil). Here, we present the detailed epidemiological and influenza vaccine effectiveness findings for the Northern Hemisphere 2014–2015 influenza season.

**Electronic supplementary material:**

The online version of this article (doi:10.1186/s12889-016-3378-1) contains supplementary material, which is available to authorized users.

## Introduction

Every year, between 5 % and 10 % of adults and 20 – 30 % of children have symptomatic influenza illness [1, 2], and 3 to 5 million individuals suffer from severe influenza, leading to 250,000 to 500,000 deaths [2–4]. Influenza illness can result in hospitalization and death, mainly among high-risk groups but also in a substantial proportion of previously healthy individuals [5]. In recent years, especially after the 2009 pandemic season, influenza surveillance has been expanded, as recommended by the World Health Organization (WHO), to include additional epidemiological data [6].

The Global Influenza Hospital Surveillance Network (GIHSN) is an international public-private collaboration initiated in 2012 by Sanofi Pasteur and the Fundación para el Fomento de la Investigación Sanitaria y Biomédica de la Comunitat Valenciana (FISABIO), a regional public health institution in Valencia, Spain. The aim of the GIHSN is to improve understanding of influenza epidemiology to better inform public health policy decisions. It is the first global network focusing exclusively on severe cases of influenza requiring hospitalization. The GIHSN runs a prospective, active surveillance, hospital-based epidemiological study to collect epidemiological and virological data for the Northern and Southern Hemispheres over several consecutive seasons. A standardised protocol and standard operating procedures are shared between sites allowing comparison and pooling of results [7]. The GIHSN is coordinated by FISABIO and is made up of several country sites affiliated with national health authorities. Each site coordinates several hospitals in its region. The network currently includes 27 hospitals coordinated by 7 sites in 6 countries (St. Petersburg and Moscow, Russian Federation; Prague, Czech Republic; Istanbul, Turkey; Beijing, China; Valencia, Spain; and Rio de Janeiro, Brazil).

The surveillance data collected by the GIHSN are used to describe the circulating strains related to severe disease, estimate the burden of severe influenza disease, and evaluate the benefit of influenza vaccination to prevent severe disease. Results have been published from the network’s first two seasons, 2012–2013 [5, 8] and 2013–2014 [9]. In this report, we describe the influenza epidemiology and vaccine effectiveness results from the GIHSN during the 2014–2015 influenza season. Complete data from the Southern Hemisphere was not available at the time of the meeting or during analysis and writing, so only data provided by sites in the Northern hemisphere during the 2014–2015 season are presented.

## Methods

### Summary of overall methodology

**Fig. 1:**
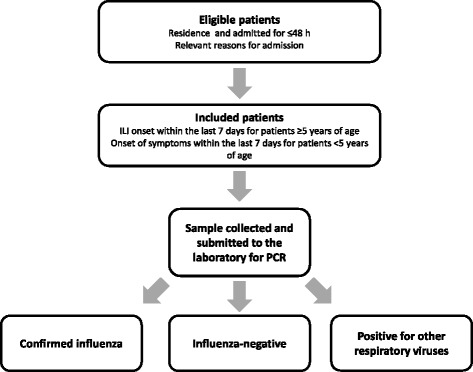
Overview of the methodology used by the GIHSN

As described in detail elsewhere [7], patients admitted in the participating hospitals are included, after written consent, if they are residents in the predefined hospital’s catchment area, present with an acute illness possibly related to influenza, are not institutionalised, and the onset of symptoms was within 7 days of admission. Swabs are collected from patients meeting the inclusion criteria and tested by reverse transcription-polymerase chain reaction (RT-PCR) for influenza (Fig. 1). Influenza-positive samples are sub-typed by RT-PCR to identify A(H1N1)pdm09, A(H3N2), B/Yamagata-lineage, and B/Victoria-lineage strains. Vaccine effectiveness is assessed using a test-negative design in which vaccine coverage is compared between admissions with and without laboratory-confirmed influenza.

### Epidemiological analysis

Epidemiological and virological data were collected from 7 coordinating sites and a total of 27 hospitals in 6 countries (Additional file 1). Briefly, eligible admissions included non-institutionalised residents in the predefined catchment areas of the participating hospitals, hospitalised in the last 48 h, and with presenting illness potentially associated with influenza (Additional file 2 and Additional file 3). The study activities were performed over influenza circulation periods defined using pre-specified criteria (Additional file 3). Nasopharyngeal swabs (all subjects), pharyngeal swabs (subjects ≥14 years) or nasal swabs (subjects <14 years) were tested by semi-quantitative RT-PCR for influenza A (subtypes H3 and H1pdm09) and B (Yamagata and Victoria lineages). The distribution of hospital admission according to RT-PCR result was described by site and risk group. Secondary outcomes included hospital admissions by subtype for influenza A(H1N1)pdm09, A(H3N2), and B-lineage, by site and risk group. The significance of differences among groups or categories was estimated by the likelihood ratio test, *t*-test, or nonparametric tests as required. A *P*-value <0.05 was considered to indicate statistical significance. To describe the major determinants for admission with influenza (vs. influenza-negative admission), a stepwise logistic regression model was fitted by including all risk factors at *P* < 0.2. Adjusted odds ratios (aORs) for RT-PCR-positive vs. RT-PCR-negative admissions in the presence of major risk factors of interest were estimated by multivariate logistic regression using minimal sufficient adjustment sets of covariates identified as confounders by causal diagrams. To account for the possible effect of study site, data were fitted to a random effects logistic regression model including site as a cluster variable. Likelihood ratio tests were used to check for the potential effect of clustering by site [10]. The adjusted effect of site in the probability of influenza with admission was estimated. Heterogeneity in the effects of risk factors by influenza strain and site were quantified using the I^2^ test. Heterogeneity was defined as an I^2^ > 50 % [11, 12]. Further details are provided elsewhere [5, 7, 8].

### Influenza vaccine effectiveness analysis

Influenza vaccine effectiveness (IVE) was estimated as (1 ˗ OR) × 100, where the OR compared the vaccine coverage rate between influenza-positive and influenza-negative patients. Patients were considered vaccinated if they had received the current season’s influenza vaccine at least 14 days before symptom onset. The types of vaccines used at each site are summarised in Additional file 4. IVE overall (irrespective of vaccine type) was determined in patients who had been swabbed within 7 days of the onset of ILI symptoms. Records for which outcome, exposure, or confounding variables were missing were excluded from the multivariate IVE analyses. The adjusted IVE was estimated by logistic regression using a random effects model with study site as a shared parameter for the pooled analysis and including week of symptom onset as a continuous variable, and age group, sex, hospitalisation in the previous 12 months, presence of chronic conditions, and smoking habits as potential confounding factors. A *P*-value <0.05 was considered to indicate statistical significance. Heterogeneity in IVE estimates was assessed using the I^2^. Potential sources of heterogeneity, including coordinating site, age, and influenza subgroup were examined in ad-hoc analyses. Heterogeneity was defined as low if I^2^ statistic <25 %, moderate if 25 – 49 %, and high if ≥50 %. Further details of the methodology are described elsewhere [8].

## Results

### Epidemiology of influenza in the GIHSN during the 2014–2015 influenza season

#### Patients included in the epidemiology analysis

Twenty thousand five hundred fifty-one eligible admissions were identified between November 16, 2014 and May 23, 2015, of which 9614 met the selection criteria and were included (Table 1). Based on RT-PCR, 2177 (23 %) were positive for influenza. Major reasons for exclusion included no ILI symptoms before admission (15 %), previous admission fewer than 30 days from the current episode (13 %), admission more than 7 days after the onset of symptoms (6 %), recruitment outside periods of continuous admissions with influenza (6 %).

**Table 1:** Selection of patients and results of RT-PCR

	St. Petersburg		Moscow		Czech Republic		Turkey		Beijing		Valencia		Total	
Category	n	%	n	%	n	%	n	%	n	%	n	%	n	%
Screened admissions	3164		1934		123		1409		1425		12,496		20,551	
Exclusion criteria														
Non resident	21	0.7	95	4.9	12	9.8	73	5.2	5	0.4	50	0.4	256	1.2
Institutionalised	14	0.4	14	0.7	2	1.6	17	1.2	2	0.1	800	6.4	849	4.1
Previous discharge <30 days	31	1.0	51	2.6	8	6.5	216	15.3	13	0.9	2283	18.3	2602	12.7
Unable to communicate	20	0.6	47	2.4	2	1.6	125	8.9	0	0.0	782	6.3	976	4.7
Not giving consent	100	3.2	32	1.7	14	11.4	47	3.3	15	1.1	504	4.0	712	3.5
No ILI symptoms ≥5 years of age	19	0.6	25	1.3	1	0.8	131	9.3	18	1.3	2903	23.2	3097	15.1
Admission within 7 days of symptoms onset	181	5.7	150	7.8	4	3.3	110	7.8	44	3.1	745	6.0	1234	6.0
Previous influenza infection	1	0.0	0	0.0	0	0.0	7	0.5	0	0.0	1	0.0	9	0.0
Onset of symptoms to swab >9 days	0	0.0	1	0.1	0	0.0	2	0.1	0	0.0	1	0.0	4	0.0
Sample inadequate	0	0.0	0	0.0	0	0.0	0	0.0	0	0.0	5	0.0	5	0.0
Sample lost	0	0.0	0	0.0	0	0.0	0	0.0	1	0.1	1	0.0	2	0.0
Recruited outside periods with continuous influenza positive admissions	31	1.0	115	5.9	1	0.8	65	4.6	178	12.5	764	6.1	1154	5.6
Included with valid laboratory results	2715	85.8	1400	72.4	79	64.2	614	43.6	1149	80.6	3657	29.3	9614	46.8
RT-PCR result														
Influenza negative	2113	77.8	966	69.0	20	25.3	543	88.4	875	76.2	2920	79.8	7437	77.4
Influenza positive	602	22.2	434	31.0	59	74.7	71	11.6	274	23.8	737	20.2	2177	22.6
Subtype and lineage^a^														
A(H1N1)pdm09	47	7.8	30	6.9	7	11.9	26	36.6	1	0.4	10	1.4	121	5.6
A(H3N2)	267	44.3	163	37.6	33	55.9	6	8.5	163	59.5	611	82.9	1243	57.1
A not subtyped	48	8.0	9	2.1	2	3.3	0	0.0	0	0.0	47	6.4	106	4.9
B/Yamagata lineage	258	42.9	175	40.3	16	27.1	0	0.0	109	39.8	65	8.8	623	28.6
B/Victoria lineage	0	0	10	2.3	0	0	0	0.0	1	0.4	0	0.0	11	0.5
B not subtyped^b^	0	0	52	12.0	2	3.4	39	54.9	0	0.0	4	0.5	97	4.5

Abbreviations: ILI, influenza-like disease; RT-PCR, reverse transcriptase-polymerase chain reaction

^a^Because there were 24 mixed infections, each involving two different influenza viruses, the sum by strain may be greater than the number of patients included with lab results. Percentages are reported by total of influenza-positive cases

^b^For Turkey and Valencia, all B not subtyped were assumed to be B/Yamagata lineage based on virus circulation at these sites. This assumption was not applied for Moscow because of a mixed pattern of influenza B circulation

### Influenza viruses identified in admissions

**Fig. 2:**
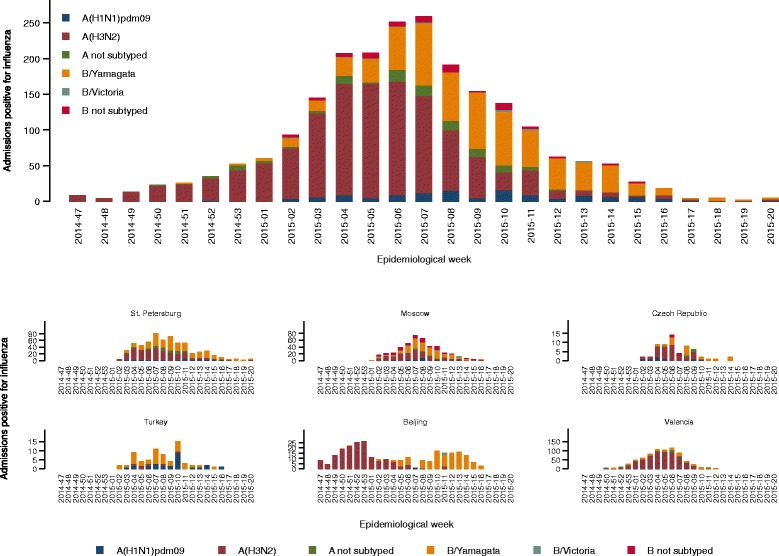
Admissions with influenza by epidemiological week and virus type, subtype, or lineage overall and by site. B strains included B not subtyped and mixed influenza infections including influenza B but excluded B/Victoria lineage

In the 2177 included influenza-positive patients, A(H3N2) (*n* = 1243; 57 %) was the most commonly identified type of influenza, followed by B/Yamagata-lineage (*n* = 623; 29 %), A(H1N1)pdm09 (*n* = 121;6 %), A not subtyped (106; 5 %), B lineage not determined (*n* = 97; 5 %), and B/Victoria-lineage (*n* = 11; 0.5 %) (Table 1 and Fig. 2a and b). Mixed influenza infections were found in 24 cases. Influenza B lineage not determined were considered B/Yamagata-lineage for 39 cases in Turkey and four in Valencia. Due to the mixed circulation of B/Yamagata and B/Victoria lineages in Moscow, this assumption was not applied at that site to cases where B-lineage was not determined.

### The 2014–2015 influenza season at the GIHSN sites

Influenza was detected over a span of 27 weeks, from week 47 of 2014 to week 20 of 2015, with the peak at week 7 of 2015 (Fig. 2). The earliest start of the influenza season was reported in Beijing, where influenza-positive admissions occurred over a span of 23 weeks in two waves, the first due to A(H3N2) and the second due to B/Yamagata-lineage (Fig. 2). The latest influenza-positive admission was in St. Petersburg, where continuous weekly admissions with influenza were observed over a span of 19 weeks.

A(H3N2) was the most frequently detected influenza virus in St. Petersburg (44 % of positives), Czech Republic (56 %), Beijing (60 %), and Valencia (83 %) (Table 1). B/Yamagata-lineage was the second-most frequently detected influenza virus in St. Petersburg (43 %), Czech Republic (27 %), and Beijing (39 %). With the exception of Beijing and Turkey, A(H3N2) and B/Yamagata-lineage co-circulated at all sites (Fig. 2). In Turkey, A(H3N2) accounted for only 8.5 % of positives, and instead, B influenza viruses predominated (55 %), followed by A(H1N1)pdm09 (37 %), with co-circulation of these two viruses (Table 1 and Fig. 2).

### Main characteristics of included patients

Overall, all age groups were represented. Approximately one-third of included admissions were patients less than 5 years of age, one-third were 5 to 64 years of age, and one-third were 65 years of age or older (Table 2). More than half of the included patients were male (*n* = 5417; 56 %). Most (*n* = 5867; 61 %) did not have an underlying chronic condition, and most (*n* = 6756; 70 %) had not been hospitalised in the 12 months before the current episode. Among the 39 % (*n* = 3747) of admissions in patients with underlying chronic conditions, the most frequent were cardiovascular disease (*n* = 1998; 21 %), chronic respiratory conditions (including chronic obstructive pulmonary disease [COPD; *n* = 1459] and asthma [*n* = 446]; 20 %), diabetes (*n* = 1048; 11 %), and renal disease (*n* = 606; 6 %). Few patients had active neoplasms (3 %), neuromuscular diseases (3 %), autoimmune diseases (2 %), liver disease (2 %), or immunodeficiency (1 %).

**Table 2:** Characteristics of included patients overall and by site

	St. Petersburg		Moscow		Czech Republic		Turkey		Beijing		Valencia		Total	
	*N* = 2715		*N* = 1400		*N* = 79		*N* = 614		*N* = 1149		*N* = 3657		*N* = 9614	
Characteristic	n	%	n	%	n	%	n	%	n	%	n	%	n	%
Age in years, median (range)	3 (0–94)		19 (0–90)		51 (19–91)		12 (0–98)		8 (0–96)		73 (0–106)		21 (0–106)	
Age group														
0–1 y	714	26.3	137	9.8	0	0.0	112	18.2	76	6.6	476	13.0	1515	15.8
2–4 y	1034	38.1	371	26.5	0	0.0	133	21.7	403	35.1	265	7.2	2206	22.9
5–17 y	357	13.1	171	12.2	0	0.0	80	13.0	147	12.8	72	2.0	827	8.6
18–49 y	426	15.7	632	45.1	38	48.1	38	6.2	106	9.2	221	6.0	1461	15.2
50–64 y	110	4.1	59	4.2	14	17.7	75	12.2	131	11.4	359	9.8	748	7.8
65–74 y	39	1.4	13	0.9	12	15.2	69	11.2	93	8.1	593	16.2	819	8.5
75–84 y	31	1.1	11	0.8	9	11.4	80	13.0	127	11.1	969	26.5	1227	12.8
≥85 y	4	0.1	6	0.4	6	7.6	27	4.4	66	5.7	702	19.2	811	8.4
Sex														
Male	1489	54.8	827	59.1	40	50.6	356	58.0	696	60.6	2009	54.9	5417	56.3
Female	1226	45.2	573	40.9	39	49.4	258	42.0	453	39.4	1648	45.1	4197	43.7
Chronic conditions														
0	2380	87.7	1246	89.0	29	36.7	182	29.6	820	71.4	1210	33.1	5867	61.0
1	244	9.0	116	8.3	31	39.2	196	31.9	233	20.3	1026	28.1	1846	19.2
>1	91	3.4	38	2.7	19	24.1	236	38.4	96	8.4	1421	38.9	1901	19.8
Previously hospitalised (last 12 months)														
No	1781	65.6	1123	80.2	56	70.9	341	55.5	964	85.1	2491	68.1	6756	70.4
Yes	934	34.4	277	19.8	23	29.1	273	44.5	169	14.9	1166	31.9	2842	29.6
Underlying chronic conditions														
Cardiovascular disease	140	5.2	68	4.9	29	36.7	215	35.0	218	19.0	1328	36.3	1998	20.8
Chronic obstructive pulmonary disease	51	1.9	19	1.4	6	7.6	153	24.9	137	11.9	1093	29.9	1459	15.2
Asthma	60	2.2	19	1.4	3	3.8	74	12.1	8	0.7	282	7.7	446	4.6
Immunodeficiency/organ transplant	30	1.1	0	0.0	3	3.8	48	7.8	0	0.0	25	0.7	106	1.1
Diabetes	32	1.2	14	1.0	12	15.2	96	15.6	34	3.0	860	23.5	1048	10.9
Renal impairment	18	0.7	26	1.9	4	5.1	61	9.9	12	1.0	485	13.3	606	6.3
Neuromuscular disease	68	2.5	15	1.1	1	1.3	79	12.9	12	1.0	92	2.5	267	2.8
Neoplasm	7	0.3	9	0.6	9	11.4	79	12.9	7	0.6	190	5.2	301	3.1
Cirrhosis/liver disease	34	1.3	21	1.5	5	6.3	19	3.1	5	0.4	118	3.2	202	2.1
Autoimmune disease	13	0.5	14	1.0	4	5.1	22	3.6	0	0.0	122	3.3	175	1.8
Pregnant (women 15–45 y)	0	0.0	291	95.7	1	7.1	1	4.8	0	0.0	5	5.7	298	45.4
Obese (all ages)	263	9.7	162	11.6	12	15.2	109	17.8	155	13.5	957	26.2	1658	17.2
Outpatient consultations last 3 months														
0	1215	44.8	492	35.1	23	29.1	113	18.4	4	0.3	649	17.7	2496	26.0
1	895	33.0	314	22.4	19	24.1	100	16.3	697	60.9	678	18.5	2703	28.1
>1	605	22.3	594	42.4	37	46.8	401	65.3	443	38.7	2330	63.7	4410	45.9
Smoking habits (patients ≥18 y)														
Never smoker	325	53.3	345	47.9	40	50.6	135	46.7	269	51.4	1363	47.9	2477	48.9
Past smoker	76	12.5	136	18.9	19	24.1	117	40.5	162	31	1034	36.4	1544	30.5
Current smoker	209	34.3	240	33.3	20	25.3	37	12.8	92	17.6	447	15.7	1045	20.6
Functional status impairment (Barthel score; patients ≥65 y)														
Total (0–15)	1	1.4	0	0.0	3	11.1	6	3.4	23	10.0	166	7.3	199	7.1
Severe (20–35)	1	1.4	0	0.0	1	3.7	3	1.7	19	8.3	71	3.1	95	3.4
Moderate (40–55)	1	1.4	1	3.3	1	3.7	6	3.4	37	16.1	140	6.2	186	6.6
Mild (60–90)	25	33.8	6	20.0	9	33.3	76	43.2	136	59.1	414	18.3	666	23.8
Minimal (95–100)	46	62.2	23	76.7	13	48.1	85	48.3	15	6.5	1473	65.1	1655	59.1
Sampling time														
0–2 days	1351	49.8	655	46.8	20	25.3	125	20.4	324	28.2	896	24.5	3371	35.1
3–4 days	915	33.7	523	37.4	26	32.9	213	34.7	382	33.2	1572	43.0	3631	37.8
5–7 days	449	16.5	221	15.8	24	30.4	239	38.9	358	31.2	1058	28.9	2349	24.4
8–9 days	0	0.0	1	0.1	9	11.4	37	6.0	85	7.4	131	3.6	263	2.7
Influenza vaccination ≥14 days from symptom onset	59	2.2	39	2.8	1	1.3	28	4.6	127	11.1	1759	48.1	2013	20.9

Just under half (*n* = 298; 45 %) of the admitted women 15–45 years of age were pregnant. Obese patients represented 17 % (*n* = 1658) of admissions. Among admissions in adult patients (≥18 years; *n* = 5066), 1045 (21 %) were current smokers, 1544 (30 %) were past smokers, and 2477 (49 %) had never smoked. Among elderly patients (≥65 years; *n* = 2857), 17 % (*n* = 480) had severe functional impairment as defined by a Barthel index <60. Finally, 2013 (21 %) admissions were in patients that had received the current season’s influenza vaccine at least 14 days before the onset of symptoms. Overall, swabs were obtained within 4 days after the onset of symptoms onset in 7002 (73 %) of included admissions.

### Site-related characteristics of included patients

Patients included in St. Petersburg were younger than patients included at other sites (Table 2). The difference in age of included patients was especially marked when comparing St. Petersburg with the Czech Republic and Valencia. Patients were most frequently young adults in Moscow and the Czech Republic. Ages were homogeneously distributed in Turkey and Beijing (*P* = 0.9480). By contrast, in Valencia most (62 %) admissions were in elderly patients (≥65 years).

Patients without comorbidities represented 88 % of admissions in St. Petersburg, 89 % in Moscow, 71 % in Beijing, 37 % in Czech Republic, 30 % in Turkey, and 33 % in Valencia. Of the different chronic conditions, cardiovascular disease, respiratory disease, and diabetes were the most common, and their relative importance at each site corresponded to the proportion of patients with one or more underlying chronic condition.

In Moscow, among admissions in patients with known risk factors for influenza, pregnant women represented the majority of admissions (*n* = 291; 96 %). Obese patients represented 10 – 15 % of admissions in St. Petersburg, Moscow, the Czech Republic, and Beijing, whereas 18 % in Turkey and 26 % in Valencia were obese. The proportion of who never smoked ranged from 47 – 53 % in adult (≥18 years) admissions and was similar across sites (*p* = 0.1520). The overall proportion of current smokers, however, differed, with the highest rate (34 %) in St. Petersburg and Moscow (33 %), followed by Czech Republic (25 %), Beijing (18 %), Valencia (16 %), and Turkey (13 %) (*p* < 0.0001). For elderly adults, functional impairment status was mild or minimal in 83–97 % of included admissions at all sites except Beijing, where 30 % of admissions in elderly patients had moderate to severe functional impairment. Rates of influenza vaccination were below 5 % for all sites except Beijing (11 %) and Valencia (48 %).

### Heterogeneity between sites

The proportion of samples with positive results differed between sites, from as low as 12 % for Turkey to as high as 75 % for the Czech Republic (Table 1; *p* < 0.0001 by test of homogeneity for equal odds). This difference persisted after excluding pregnant women and excluding the two sites with extreme results: proportions with positive results were 22 % for St. Petersburg, 31 % for Moscow, 24 % for Beijing, and 20 % for Valencia (*p* < 0.0014 by test of homogeneity for equal odds). After excluding pregnant women, however, proportions were homogenous in St. Petersburg, Moscow, and Beijing (*p* < 0.1464 by test of homogeneity for equal odds). After adjusting for sex, age, comorbidity, previous admissions, time to swab, influenza vaccination, and calendar time, the heterogeneity of aORs for a positive result were similar to the unadjusted results (Additional file 5 and Additional file 6; I^2^ = 96.4 %; *p* <0.0001).

### Risk of admission with influenza according to age and sex and variability by influenza virus

**Fig. 3:**
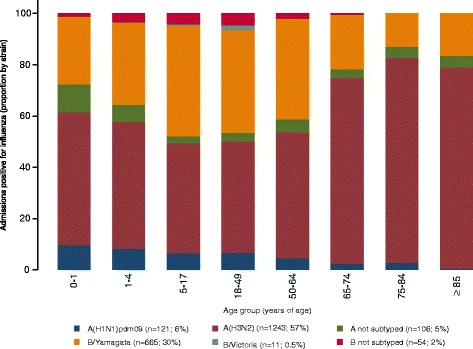
Proportion of admissions by strain and age group

Influenza positivity was related to age. Overall, influenza-positive admissions tended to be older than influenza-negative admissions (Table 3). Admissions positive for A(H1N1)pdm09 were younger than those negative for influenza, those positive for A(H3N2), and those positive for B/Yamagata-lineage. Also, admissions positive for A(H3N2) were older than influenza-negative admissions, those positives for A(H1N1)pdm09, and those positive for B/Yamagata-lineage (Table 3 and Fig. 3).

**Table 3:** Characteristics of included patients according to PCR result

	Influenza-negative		Influenza-positive			A(H1N1)pdm09			A(H3N2)			B/Yamagata lineage		
	*N* = 7437		*N* = 2177			*N* = 115			*N* = 1231			*N* = 646		
	n	%	n	%	P vs. negative	n	%	P vs. negative	n	%	P vs. negative	n	%	P vs. negative
Age in years, median (range)	18.4 (0–106)		32.8 (0–100)		0.0001	5.6 (0–85)		0.0861	54.5 (0–100)		0.0001	26.2 (0–96)		0.0013
Age group					<0.0001			<0.0001			<0.0001			<0.0001
0–1 y	1371	18.4	144	6.6		14	12.2		74	6.0		37	5.7	
2–4 y	1777	23.9	429	19.7		36	31.3		212	17.2		133	20.6	
5–17 y	547	7.4	280	12.9		14	12.2		121	9.8		118	18.3	
18–49 y	1038	14	423	19.4		28	24.3		183	14.9		168	26.0	
50–64 y	557	7.5	191	8.8		9	7.8		92	7.5		73	11.3	
65–74 y	608	8.2	211	9.7		5	4.3		153	12.4		45	7.0	
75–84 y	933	12.5	294	13.5		8	7.0		235	19.1		38	5.9	
≥85	606	8.1	205	9.4		1	0.9		161	13.1		34	5.3	
Sex					<0.0001			0.0390			0.0003			0.0040
Male	4276	57.5	1141	52.4		55	47.8		651	52.9		333	51.5	
Female	3161	42.5	1036	47.6		60	52.2		580	47.1		313	48.5	
Chronic conditions					0.0940			0.1600			<0.0001			0.004
0	4572	61.5	1295	59.5		78	67.8		643	52.2		434	67.2	
≥1	2865	38.5	882	40.5		37	32.2		588	47.8		212	32.8	
Underlying chronic conditions														
Cardiovascular disease	1529	20.6	469	21.5	0.3210	18	15.7	0.1820	319	25.9	<0.0001	109	16.9	0.02200
Chronic obstructive pulmonary disease	1153	15.5	306	14.1	0.0570	7	6.1	0.0001	222	18.0	0.0270	60	9.3	<0.0001
Asthma	346	4.7	100	4.6	0.9080	5	4.3	0.8760	65	5.3	0.3440	24	3.7	0.2600
Immunodeficiency/organ transplant	92	1.2	14	0.6	0.0130	0	0.0	0.0920	8	0.6	0.0550	6	0.9	0.4750
Diabetes	814	10.9	234	10.7	0.7960	8	7.0	0.1480	180	14.6	<0.0001	34	5.3	<0.0001
Renal impairment	463	6.2	143	6.6	0.5640	2	1.7	0.0200	108	8.8	0.0010	27	4.2	0.0280
Neuromuscular disease	215	2.9	52	2.4	0.2020	8	7.0	0.0290	19	1.5	0.0040	22	3.4	0.4670
Neoplasm	238	3.2	63	2.9	0.4660	4	3.5	0.8680	38	3.1	0.8330	17	2.6	0.4160
Cirrhosis/liver disease	168	2.3	34	1.6	0.0390	0	0.0	0.0220	19	1.5	0.0950	11	1.7	0.3390
Autoimmune disease	127	1.7	48	2.2	0.1360	1	0.9	0.4470	36	2.9	0.0060	8	1.2	0.3510
Pregnant (women 15–45 y)	138	33.7	160	64.8	0.0000	11	68.8	0.0050	64	55.7	<0.0001	68	70.1	<0.0001
Obese (all ages)	1300	17.5	358	16.4	0.2590	15	13.0	0.1970	223	18.1	0.5890	92	14.2	0.0320
Smoking habits (patients ≥18 y)														
Never smoked	1760	47.0	717	54.2		23	45.1		451	54.7		191	53.4	
Past smoker	1164	31.1	380	28.7		14	27.5		252	30.6		96	26.8	
Current smoker	818	21.9	227	17.1		14	27.5		121	14.7		71	19.8	
Functional status impairment (Barthel score; patients ≥65 y)														
Total (0–15)	152	7.2	47	6.7		0	0.0		41	7.6		5	4.3	
Severe (20–35)	76	3.6	19	2.7		0	0.0		13	2.4		4	3.5	
Moderate (40–55)	140	6.7	46	6.6		0	0.0		32	5.9		11	9.6	
Mild (60–90)	518	24.7	148	21.1		1	7.1		111	20.5		32	27.8	
Minimal (95–100)	1214	57.8	441	62.9		13	92.9		345	63.7		63	54.8	
Influenza vaccination ≥14 d since onset of symptoms	1566	21.1	447	20.5	0.5960	6	5.2	<0.0001	356	28.9	<0.0001	57	8.8	<0.0001

Not subtyped A and B were 24 patients with mixed influenza infections were not included in the analysis by strain

After adjusting for sex, occupational class, comorbidity, influenza vaccination, time to swab, and the clustering effect of site, heterogeneity due to strain was significant for admissions in subjects ≥5 years of age due to a decrease in aOR with age for admission with A(H1N1)pdm09 (Table 4 and Additional file 7). After excluding admissions with A(H1N1)pdm09, the aOR for admission with influenza was homogeneous for elderly patients but heterogeneous for patients 5–64 years of age (I^2^ = 75–77 %) due to a higher aOR for admissions with B/Yamagata-lineage than for A(H3N2) (Additional file 7).

**Table 4:** Subject characteristics and risk of admission with influenza

	All admissions	Influenza-positive		Crude OR		Heterogeneity by strain (I^2^)^a^	aOR^b^	
	*N* = 9164	*N* = 2177						
Characteristic	n	n	%	Value	95 % CI		Value	95 % CI
Age								
0–1 y	1515	144	9.5	1.00	-	32.5 %	1.00	-
2–4 y	2206	429	19.4	2.30	1.88-2.81	0.0 %	2.14	1.74-2.64
5–17 y	827	280	33.9	4.87	3.90-6.10	73.3 %	4.34	3.42-5.51
18–49 y	1461	423	29.0	3.88	3.16-4.77	59.2 %	3.11	2.49-3.90
50–64 y	748	191	25.5	3.26	2.57-4.14	72.5 %	4.08	3.11-5.36
65–74 y	819	211	25.8	3.30	2.62-4.17	67.8 %	4.99	3.76-6.64
75–84 y	1227	294	24.0	3.00	2.42-3.72	62.4 %	4.51	3.43-5.92
≥85	811	205	25.3	3.22	2.55-4.07	71.2 %	4.79	3.59-6.40
Sex^c^								
Male	5417	1141	21.1	1.00	-	0.0 %	1.00	-
Female	4197	1036	24.7	1.24	1.13-1.37	0.0 %	1.21	1.09-1.34
Female non-pregnant	3899	876	22.5	1.11	1.00-1.22	0.0 %	1.10	0.99-1.23
Other risk factors (excludes pregnant women)								
Comorbidity^d^	3709	856	23.1	1.15	1.04-1.27	62.8 %	1.48	1.30-1.69
Cardiovascular disease	1996	468	23.4	1.17	1.04-1.33	22.2 %	1.47	1.25-1.72
Chronic obstructive pulmonary disease	1458	306	21.0	1.02	0.88-1.17	57.6 %	1.39	1.15-1.68
Asthma	440	96	21.8	1.07	0.84-1.35	0.0 %	1.37	1.04-1.80
Immunosuppression	106	14	13.2	0.58	0.33-1.03	0.0 %	0.76	0.40-1.46
Diabetes	1048	234	22.3	1.10	0.94-1.29	0.0 %	1.36	1.10-1.70
Renal disease	588	129	21.9	1.08	0.88-1.32	48.2 %	1.23	0.95-1.59
Neuromuscular	167	52	31.1	0.93	0.68-1.26	38.9 %	1.13	0.80-1.58
Neoplasm (active)	301	63	20.9	1.01	0.76-1.35	55.6 %	1.29	0.92-1.81
Liver disease	200	33	16.5	0.76	0.52-1.11	50.8 %	0.79	0.52-1.21
Autoimmune disease	161	39	24.2	1.22	0.85-1.77	0.0 %	1.44	0.95-2.18
Obese^e^	1620	337	20.8	1.0	0.9-1.2	0.0 %	0.87	0.73-1.03
Pregnancy^f^	298	160	53.7	3.45	2.23-5.34	0.0 %	2.08	1.43-3.03
Associated comorbidity	38	26	68.4	7.07	3.09-16.18	0.0 %	4.29	2.65-6.94
No comorbidity	260	126	48.5	3.05	2.08-4.47	0.0 %	1.80	1.22-2.66

^a^Strains considered: A(H3N2), A(H1N1)pdm09 and B/Yamagata

^b^Minimal sufficient adjustment sets for estimating the exposure or risk factor effect on the risk of admission with influenza vs. all included admissions without underlying conditions or pregnant

^c^Female or female non-pregnant vs. male. aORs were adjusted for age, occupational social class group, underlying comorbidity, obesity, influenza vaccination, time to swab, calendar time, and site as a clustering factor

^d^One or more underlying conditions or individual comorbidities vs. no comorbidity. aORs were adjusted for sex, occupational social class group, obesity, influenza vaccination, time to swab, calendar time, and site as a clustering factor

^e^aOR adjusted for sex, age, occupational social class group, influenza vaccination, time to swab, calendar time, and site as a clustering factor

^f^Women 15-45 years of age included in Moscow, St. Petersburg, Czech Republic, Turkey and Valencia. aOR adjusted for smoking habits, time to swab, calendar time, comorbidity, and site as a clustering factor. For results stratified by comorbidity, aORs were adjusted by the same covariates and were estimated taking into account the interaction between pregnancy and comorbidity

Female patients had a higher risk than male patients of being influenza-positive (aOR, 1.21 [95 % CI, 1.09-1.34]), irrespective of strain (I^2^ = 0 %). However, after excluding pregnant women, the risk was more similar for males and females (aOR, 1.10 [95 % CI, 0.99–1.23]) (Table 4).

### Risk of admission with influenza according presence of comorbidity

Similar proportions of influenza-positive admissions (882/2177; 41 %) and influenza-negative admissions (2865/7437; 39 %) had one or more chronic underlying condition (*p* = 0.0940) (Table 3). After excluding pregnant women, 42 % of influenza-positive admissions had comorbidity compared to 39 % of influenza-negative admissions (*p* = 0.006) (data not shown). The aOR for admission with influenza was 1.5 (95 % CI, 1.3–1.7) for patients with comorbidities, although the values were heterogeneous by strain (I^2^ = 63 %) (Table 4) due to a higher aOR for admission with A(H3N2) or B/Yamagata-lineage in patients with comorbidities compared to patients with no underlying conditions (Additional file 8).

**Fig. 4:**
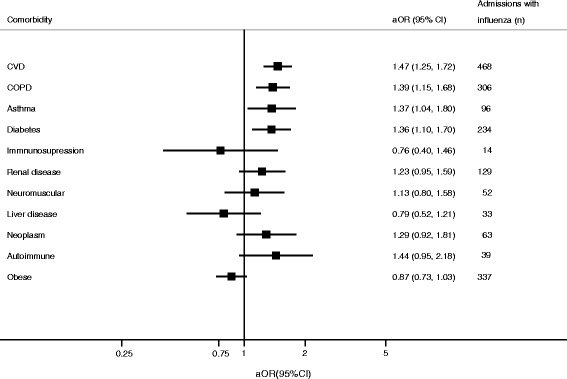
Adjusted odds ratio (aOR) and number of admissions with influenza according to comorbidity. CVD, cardiovascular disease. COPD: chronic obsructive pulmonary disease

Irrespective of the involved strain (I^2^ = 22 %), the risk of admission with influenza was significantly increased in patients with cardiovascular disease (aOR = 1.5 [95 % CI, 1.3–1.7), asthma (1.4 [95 % CI, 1.0–1.8]), or diabetes (1.4 [95 % CI, 1.1–1.7]) (Table 4, Fig. 4, and Additional file 9). The aOR was heterogeneous for the risk of admission with influenza in patients with COPD (aOR 1.4 [95 % CI, 1.2–1.7]; I^2^ = 58 %) due to lower aOR for admission with A(H1N1)pdm09 (Additional file 9). Point values for aORs were above 1.0 for admission with influenza for patients with renal, neuromuscular, or autoimmune disease, but 95 % CIs overlapped 1.0. In patients with active neoplasms, the overall aOR for influenza-positive admission was heterogeneous and not significant (I^2^ = 56 %; aOR = 1.3 [95 % CI, 0.9–1.8]), although for B/Yamagata-lineage, the risk was significantly elevated (aOR = 2.2 [95 % CI, 1.1–4.1]) (Additional file 9).

### Risk of admission with influenza according pregnancy

A total of 298 included admissions were pregnant women 15–45 years of age, 291 of whom were included in Moscow, one in the Czech Republic, one in Turkey, and five in Valencia (Table 2). No pregnant women were included in Beijing. Non-pregnant women in this age group accounted for another 359 included admissions, of which 197 were in St. Petersburg, 13 in Moscow, 13 in the Czech Republic, 20 in Turkey, 33 in Beijing, and 83 in Valencia (data not shown).

The probability of laboratory-confirmed influenza was higher in included pregnant women than included same age non-pregnant women (54 % vs. 24 %; *p* < 0.0001; data not shown). After taking into account clustering by site (and not considering data from Beijing), the crude OR of admission with influenza was 3.5 (95 % CI, 2.2–5.3) (Table 4). This crude estimated OR was higher in pregnant women with associated comorbidity (OR 7.1 [95 % CI, 3.1–16.2]), with moderate evidence of an interaction between comorbidity and pregnancy before adjustment (*p* = 0.0659) and a significant interaction after adjustment (*p* < 0.0001). Taking into account the modifying effect of associated comorbidity, the aOR for admission with influenza in pregnant women was 4.3 (95 % CI, 2.7–6.9) in presence of associated comorbidity and 2.1 (95 % CI, 1.4–3.0) for pregnant women with no comorbidity. In both cases, the values were homogenous (I^2^ = 0 %) for A(H3N2), A(H1N1)pdm09, and B/Yamagata-lineage infections.

**Fig. 5:**
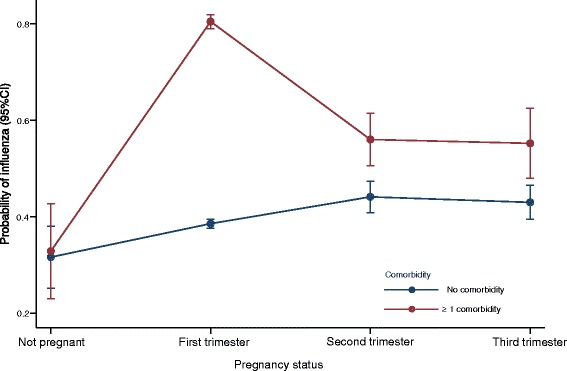
Predicted probability of admission with influenza in non-pregnant 15 – 45 years old women and by pregnancy trimester in same age pregnant women

The probability of admission with influenza was higher in all three trimesters for pregnant women without associated comorbidities than for non-pregnant women in the same age group without comorbidity. In pregnant women with comorbidities, the risk of admission with influenza was highest in the first trimester (Fig. 5 and Additional file 10).

### Risk of admission with influenza and complications by strain

Intensive care unit (ICU) admissions, extracorporeal membrane oxygenation, and mechanical ventilation were more frequent for influenza-negative than for influenza-positive admissions (*p* ≤ 0.002), whereas rates of in-hospital death were similar (*p* = 0.3460) (Table 5). By strain, the point estimate of rates of ICU admission and extracorporeal membrane oxygenation were higher in admissions with A(H1N1)pdm09, although differences were not significant. In contrast, rates of in-hospital death were significantly higher in admissions with A(H3N2) (*p* = 0.0080). Less than 4 % of admissions in these categories experienced a severe outcome. Finally, length of stay did not differ between influenza-positives and influenza-negative admissions for influenza overall or between strains (Table 5).

**Table 5:** Influenza severity and complications by RT-PCR result

Category	Influenza-negative		Influenza-positive		*P*-value influenza-negative vs. positive	A(H1N1)pdm09		A(H3N2)		B/Yamagata lineage		*P*-value for distribution by strain
	*N* = 7437		*N* = 2177			*N* = 115		*N* = 1231		*N* = 646		
	n	%	n	%		n	%	n	%	n	%	
Severity indicator												
Intensive care unit admission	184	2.5	31	1.4	0.0020	4	3.5	15	1.2	9	1.4	0.2400
Mechanical ventilation	123	1.7	20	0.9	<0.0001	1	0.9	14	1.1	4	0.6	0.5230
Extracorporeal membrane oxygenation	184	2.8	25	1.3	0.0020	3	2.6	9	0.8	9	1.7	0.1600
Death during hospitalisation	131	1.8	32	1.5	0.3460	1	0.9	26	2.1	3	0.5	0.0080
Length of stay (days), median (interquartile range)	6	(4-9)	6	(4-8)	0.0612	6	(3-8)	6	(3-8)	6	(4-8)	0.2835
Pulmonary complications					<0.0001							<0.0001
None	1939	26.1	1212	55.7		53	46.1	697	56.6	353	54.6	
Pneumonia	1545	20.8	364	16.7		18	15.7	214	17.4	109	16.9	
COPD exacerbation	265	3.6	87	4.0		3	2.6	66	5.4	15	2.3	
Respiratory failure	55	0.7	32	1.5		0	0.0	23	1.9	5	0.8	
Asthma exacerbation	28	0.4	12	0.6		0	0.0	11	0.9	1	0.2	
pulmonary collapse	5	0.1	1	0.0		0	0.0	1	0.1	0	0.0	
Acute respiratory distress syndrome	7	0.1	2	0.1		0	0.0	2	0.2	0	0.0	
Bronchiolitis	416	5.6	201	9.2		15	13.0	91	7.4	75	11.6	
Upper respiratory infection	3172	42.7	266	12.2		26	22.6	126	10.2	88	13.6	
Metabolic failure					0.4690							0.3530
Acute renal failure	87	1.2	32	1.5		1	0.9	24	1.9	5	0.8	
Diabetic coma	4	0.1	2	0.1		0	0.0	1	0.1	0	0.0	
Fluid/electrolyte/acid-base/balance disorders	80	1.1	29	1.3		1	0.9	19	1.5	7	1.1	
Cardiovascular events					0.3390							<0.0001
None	6335	85.2	1883	86.5		107	93.0	991	80.5	612	94.7	
Acute myocardial infarction	8	0.1	5	0.2		0	0.0	5	0.4	0	0.0	
Acute heart failure	1	0.0	1	0.0		0	0.0	1	0.1	0	0.0	
Cardiac arrest	4	0.1	3	0.1		0	0.0	3	0.2	0	0.0	
Malignant hypertension	37	0.5	10	0.5		0	0.0	9	0.7	1	0.2	
Any cardiovascular condition	1050	14.1	275	12.6		8	7.0	222	18.0	33	5.0	
Systemic inflammatory response syndrome, shock, or disseminated intravascular coagulation	76	1.0	12	0.6	0.0320	2	1.7	9	0.7	1	0.2	0.0810
Neurologic events												
No	7423	99.8	2173	99.8	0.3140	114	99.1	1228	99.8	646	100.0	0.1249
Altered mental status	10	0.1	4	0.2		1	0.9	3	0.2	0	0.0	
Convulsions	4	0.1	0	0.0		0	0.0	0	0.0	0	0.0	
Major discharge diagnoses					<0.0001							<0.0001
Influenza	124	1.7	1266	58.2		76	66.1	603	49.0	456	70.6	
Pneumonia	1807	24.3	223	10.2		10	8.7	145	11.8	59	9.1	
Other respiratory disease	3653	49.1	290	13.3		20	17.4	188	15.3	57	8.8	
Cardiovascular	603	8.1	117	5.4		0	0.0	105	8.5	7	1.1	
Other	1250	16.8	281	12.9		9	7.8	190	15.4	67	10.4	

Exacerbation of chronic obstructive pulmonary disease, respiratory failure, exacerbation of asthma, and bronchiolitis were more frequently reported for influenza-positive admissions than for influenza-negative admissions (Table 5). These were associated with A(H3N2), except in the case of bronchiolitis, where the proportions for admission with all three strains (A(H3N2), A(H1N1)pdm09, and B/Yamagata-lineage) were higher than the proportion for influenza-negative admissions. Cardiovascular events were more frequently reported for admissions with influenza A(H3N2) than for admissions with influenza A(H1N1)pdm09 or B (OR 1.3 [95 % CI, 1.1–1.6]; *p* = 0.0004; data not shown), whereas, shock was more frequent in admissions with influenza A(H1N1)pdm09 (adjusted *p* < 0.0001; Table 5).

**Fig. 6:**
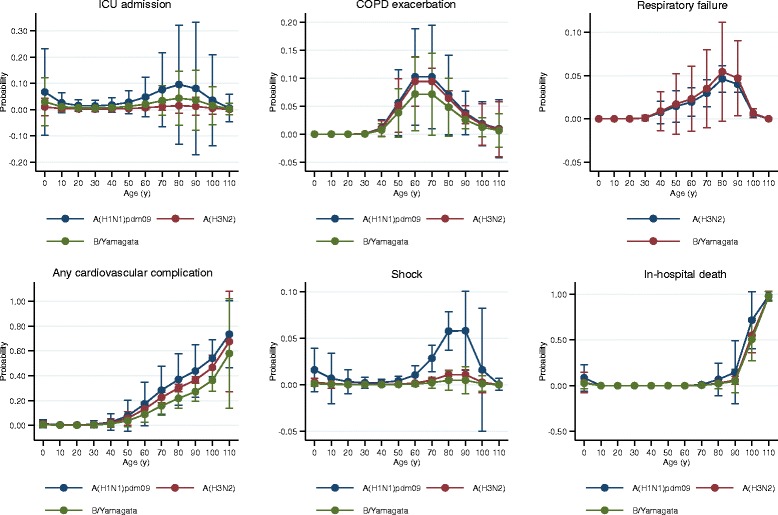
Predicted probability of severe outcome by strain (not subtyped, mixed influenza with influenza infections and B/Victoria lineage excluded)

Figure 6 shows the estimated marginal probabilities by strain and age for severe outcomes after adjusting by sex, comorbidity, calendar time, age, and clustering by site. We found several non-significant associations: A(H1N1)pdm09 was associated with intensive care unit admission and shock; A(H3N2) was associated with an increased probability of COPD exacerbation, respiratory failure, cardiovascular complications, and death; B/Yamagata-lineage was related to respiratory failure; and all three strains were related to death at both extremes of age (Fig. 6). We found similar non-significant associations for complications when influenza-negative admissions were included (Additional file 11).

### Influenza vaccine effectiveness in the GIHSN during the 2014–2015 influenza season

#### Patients included in the influenza vaccine effectiveness analysis

After applying exclusions related to vaccine contraindication (egg allergy and <6 months of age), 8455 specimens collected from November, 2014 through May, 2015 were included in the IVE analyses. Of all collected specimens, 2027 (24 %) were positive for influenza, of which 1165 (57 %) were positive for A(H3N2), 104 (5 %) for A(H1N1)pdm09, and 625 (31 %) for B Yamagata-lineage (Table 6). Overall, 22 % (*n* = 446) of influenza-positive admissions and 24 % (*n* = 1556) of influenza-negative admissions were vaccinated (*p* = 0.042) (Table 7). The proportion of patients vaccinated with the seasonal influenza vaccine in 2014–2015 ≥ 14 days before symptom onset was 3 % in St. Petersburg (*n* = 43) and Moscow (*n* = 30), 5 % in Turkey (*n* = 22), 11 % (*n* = 94) in Beijing, and 54 % (*n* = 1367) in Valencia (data not shown).

**Table 6:** IVE for all cases and for targeted groups only by age and strain

			Influenza-positive		Influenza-negative		Crude IVE		Adjusted IVE	
Population	Strain	Age	Total	Vaccinated	Total	Vaccinated	Percent (95 % CI)	P interaction	Percent (95 % CI)	P interaction
Overall	Any	Any	2027	446	6428	1556	-1 (-17, 12)		22 (8, 33)	
		<65 y	1334	78	4299	289	-4 (-36, 20)	0.090	-5 (-38, 20)	0.054
		≥65 y	693	368	2129	1267	21 (5, 34)		24 (9, 37)	
	A(H3N2)	Any	1165	356	6428	1556	-6 (-24, 10)		20 (4, 33)	
		<65 y	630	50	4299	289	-15 (-59, 17)	0.036	-16 (-64, 17)	0.031
		≥65 y	535	306	2129	1267	24 (7, 37)		25 (8, 39)	
	A(H1N1)	Any	104	7	6428	1556	25 (-85, 69)		27 (-82, 71)	
		<65 y	91	3	4299	289	16 (-173, 74)	0.996	21 (-161, 76)	0.993
		≥65 y	13	4	2129	1267	47 (-128, 88)		59 (-83, 91)	
	B/Yamagata	Any	625	57	6428	1556	16 (-17, 39)		31 (2, 52)	
		<65 y	509	20	4299	289	7 (-51, 42)	0.266	29 (-17, 58)	0.273
		≥65 y	116	37	2129	1267	38 (-2, 62		33 (-12, 61)	
Targeted groups only	Any	Any	1670	425	5077	1462	13 (-2, 26)		23 (8, 35)	
		<65 y	977	57	2948	195	-21 (-70. 14)	0.037	-12 (-58, 20)	0.019
		≥65 y	693	368	2129	1267	26 (16, 42)		28 (14, 41)	
	H3N2	Any	994	344	5077	1462	13 (-4, 28)		22 (5, 36)	
		<65 y	459	38	2948	195	-12 (-65, 24)	0.051	-20 (-80. 21)	0.030
		≥65 y	535	306	2129	1267	27 (11, 41)		28 (11, 42)	
	H1N1	Any	84	6	5077	1462	44 (-58, 80)		46 (-52, 81)	
		<65 y	71	2	2948	195	33 (-198, 85)	0.793	39 (-167, 86)	0.770
		≥65 y	13	4	2129	1267	47 (-128, 88)		50 (-111, 89)	
	B/Yamagata	Any	486	49	5077	1462	21 (-18, 46)		30 (-5, 53)	
		<65 y	370	12	2948	195	-8 (-105, 44)	0.139	8 (-79, 53)	0.250
		≥65 y	116	37	2129	1267	38 (-2, 62)		33 (-12, 60)	

*Abbreviation*: *IVE* influenza vaccine effectiveness

**Table 7:** Characteristics of patients included in the primary analysis by vaccination status

Risk variables	Category	Unvaccinated		Vaccinated		*P* value
		n	%	N	%	
Number of patients, n (%)	Controls	4872	75.5	1556	77.7	0.042
	Cases	1581	24.5	446	22.3	
Age (y)	Median (range)	14.6 (0.8-84.0)		78.7 (9.0-91.9)		<0.001
Age group, n (%)	6–11 mo	496	7.7	3	0.1	
	1–4 y	2120	32.9	49	2.4	
	5–17 y	712	11.0	102	5.1	
	18–49 y	1347	20.9	75	3.7	
	50–64 y	591	9.2	138	6.9	
	65–74 y	388	6.0	416	20.8	
	75–84 y	512	7.9	704	35.2	
	≥85 y	287	4.4	515	25.7	
Female, n (%)	-	2825	43.8	843	42.1	0.188
Comorbidities, n (%)	None	4505	70	366	18.3	<0.001
	1	1077	16.7	647	32.3	
	>1	871	13.5	989	49.4	
Pregnant, n (%)	-	294	4.6	3	0.1	<0.001
Obesity, n (%)	-	982	15.2	564	28.2	<0.001
Morbid obesity, n (%)	-	86	1.3	54	2.7	<0.001
Previous hospitalisation within 12 months, n (%)	-	1887	29.2	761	38	<0.001
GP visit within 3 months, n (%)	None	1815	28.1	343	17.1	<0.001
	1	2027	31.4	389	19.4	
	>1	2606	40.4	1272	63.5	
Smoking, n (%)	Current	1527	23.7	225	11.2	<0.001
	Past	1069	16.6	742	37.1	
	Never	3856	59.8	1035	51.7	
Functional impairment in ≥65 y, n (%)	None or minimal	619	54.2	1021	62.4	<0.001
	Mild	324	28.4	330	20.2	
	Moderate	95	8.3	88	5.4	
	Severe	32	2.8	62	3.8	
	Total	72	6.3	123	7.5	
Sampling interval (days)	Median (range)	3 (1-7)		4(1-7)		<0.001
Sampling interval, n (%)	≤4 d	3703	57.4	990	49.5	<0.001
	5-7 d	2587	40.1	936	46.8	
	8-9 d	163	2.5	76	3.8	
Site, n (%)	St. Petersburg	2138	33.1	59	2.9	<0.001
	Moscow	1306	20.2	39	1.9	
	Turkey	503	7.8	26	1.3	
	Beijing	996	15.4	127	6.3	
	Valencia	1510	23.4	1751	87.5	
Vaccinated, n (%)						
	In 2012–2013	473	7.5	1471	73.3	<0.001
	In 2013–2014	513	8.1	1722	87.1	<0.001

Overall, 1709 of 2002 (85 %) influenza vaccinations among study patients were both self-reported and confirmed from registries. Self-report captured 156 of 2002 vaccinations (8 % overall, 67 % in Moscow, 8 % in Turkey, 1 % in Beijing, and 7 % in Valencia; data not shown). Another 137 patients (7 % overall, 12 % in St. Petersburg, 42 % in Turkey, and 7 % in Valencia; data not shown) with vaccination records failed to self-report vaccination.

The proportion of participants with comorbidity was significantly higher in vaccinated than in non-vaccinated admissions (82 % vs. 30 %, *p* < 0.001) (Table 7). Vaccination was also more common among elderly (median age = 79 years for vaccinated patients vs. 15 years for non- vaccinated patients, *p* < 0.001), obese patients (28 % obese for vaccinated patients vs. 15 % for non-vaccinated patients, *p* < 0.001), elderly patients with impairment or minimal functional impairment (28 % impaired for vaccinated patients vs. 15 % for non-vaccinated patients, *p* < 0.001), patients with outpatient visits (83 % for vaccinated patients vs. 72 % for non-vaccinated patients, *p* < 0.001), and patients admitted to a hospital in the previous 12 months (38 % for vaccinated patients vs. 29 % for non-vaccinated patients, *p* < 0.001) (Table 7). Three (0.1 %) pregnant women had received the current season’s vaccine.

Most patients vaccinated in 2014–2015 reported prior vaccination: 87 % of vaccinated patients had received the 2013–2014 vaccine (*p* < 0.001) and 73 % had received the 2012–2013 vaccine (*p* < 0.001) (Table 7). Both the 2011-2012 and 2012–2013 vaccines were received by 90 % (26/29) of cases and 77 % (89/116) of controls (*p* = 0.12).

### Influenza vaccine effectiveness

Against all-age influenza-related hospitalisation, the overall crude IVE was −1 % (95 % confidence interval [CI], −17–12), and the adjusted IVE was 22 % (95 % CI, 8–33) (Table 6). Age at admission, presence of comorbidities, and degree of functional impairment were the covariates with the largest confounding effect on crude IVE (data not shown), raising the crude IVE with adjustment.

The adjusted IVE for patients of all ages was higher against influenza B (31 % [95 % CI, 2–52]) than for influenza A(H3N2) (20 % [95 % CI, 4–33]) and influenza A(H1N1)pdm09 (27 % [95 % CI, −82–71]) (Table 6), although confidence intervals overlapped (I^2^ for adjusted IVE across strains = 0 %, *p* = 0.762; data not shown).

Age-specific estimates suggested that vaccination against any influenza was less effective in patients <65 years of age (IVE [95 % CI] = −5 % [−38–20]) than in patients ≥65 years of age (IVE = 24 % [95 % CI, 9–37]) (*P* value for effect modification of age = 0.054). This pattern of lower IVE in the younger patients was consistent across strains, but only age-specific estimates for A(H3N2) were significantly different (Table 6).

Estimates were similar when the analyses were restricted to patients belonging to the target group for vaccination (crude IVE against overall influenza for all ages = 13 % [95 % CI, −2–26], adjusted IVE = 23 % [95 % CI, 8–35]) (Table 6).

IVE estimates were consistently higher for recipients of the 2012–2013 influenza vaccine, the 2013–2014 influenza vaccine, or both vaccines than for recipients of only the current season’s vaccine, although confidence intervals overlapped (Additional file 12).

Statistical heterogeneity across sites in the estimates of IVE against influenza-related hospitalisation was relatively low, with site-specific adjusted point estimates ranging from -27 – 35 % [I^2^ = 0 %; *P* = 0.835) (Additional file 13).

Sensitivity analyses were performed to assess the effects of excluding pregnant women, participants vaccinated within 14 days before symptom onset, and without medical vaccination records. In all cases, IVE estimates remained similar to those of primary analysis (Additional file 14). Further sensitivity analyses using various statistical methods to account for potential data clustering by site showed consistent results, with no evidence of heterogeneity (I^2^ = 0 %) in estimates of IVE across methods (Additional file 15).

## Discussion

According to data collected by active surveillance within the GIHSN sites, the 2014–2015 influenza season was characterised by a predominance of A(H3N2) and B/Yamagata-lineage, and to a lesser extent, A(H1N1)pdm09, while B/Victoria-lineage was relatively rare. Reports of severe influenza, defined as hospitalisation with laboratory (i.e., PCR)-confirmed influenza, spanned 6 months and affected all ages, although influenza-related admissions were most common in older individuals. Among patients with laboratory-confirmed influenza, those with A(H1N1)pdm09 were younger than those with A(H3N2) or B/Yamagata-lineage, whereas those with B/Yamagata-lineage were most frequently young and middle-aged adults. This pattern of influenza circulation is consistent with that reported by the WHO [13]. Likewise, the age distribution of the A(H1N1)pdm09, A(H3N2) and B/Yamagata-lineage strains agrees with others’ reports [14, 15].

According to our data, comorbidity increased the risk of admission with influenza, irrespective of the strain involved. This was also the case for pregnant women. Furthermore, the combination of pregnancy and comorbidity increased the risk of admission several-fold, suggesting an interaction. Remarkably, however, nearly 60 % of eligible admissions with influenza were patients without known risk factors.

The probability of ICU admission and shock were higher in patients infected with A(H1N1)pdm09 than with other strains. Also, A(H3N2) infection was associated with respiratory failure and cardiac complications, whereas B/Yamagata-lineage was associated with an increased probability of respiratory failure. Influenza infection overall was associated with in-hospital death at both age extremes. These findings agree with other reports [15–17], although there may be differences in the absolute percentage of admissions with influenza in patients with comorbidity, patterns of severity, lengths of hospital stay, rates of ICU admission, use of supportive measures, or estimates of in-hospital death rates [15, 18, 19].

Although vaccination coverage was low at the participating sites (2.8–48 %; average 20.9 %), we found that vaccination conferred a low to moderate protective effect (adjusted IVE = 22 %). This protective effect was greater for adults ≥65 years of age than for adults <65 years of age and was greater for B/Yamagata-lineage than for A(H3N2).

The low influenza vaccine effectiveness for the 2014–2015 season is similar to others’ reports and appears to be due mostly to a mismatch between the main A(H3N2) circulating strain and the vaccine strain [20–23]. Across all strains, the IVE was lower in young patients, although only age-specific estimates for A(H3N2) were significantly different due to few cases of B/Yamagata-lineage and A(H1N1)pdm09 and a higher IVE in patients vaccinated during the 2012–2013, 2013–2014, or both seasons than in those vaccinated during the 2014–2015 season, a finding also reported by others [24]. This lower IVE in young patients, however, contrasts with previous reports where the opposite was found [25]. Thus, there appears to be variability in the interference or protection conferred by vaccination in previous seasons. This could be explained by the differences between the various strains circulating in different seasons and their distance from the vaccine strains, combined with inhibition of the immunological response when the vaccine strains are similar to those in previous seasons’ vaccines [26].

### Limitations and considerations

Our results are to be interpreted with caution due to the heterogeneity and bias of multi-centric observational studies. We assumed heterogeneity in the circulating strains, socio-demographic diverse populations observed, their health care seeking behaviour, the characteristics of the different health care systems involved, the types of participating hospitals, and by calendar time along the season. We took account of this heterogeneity by thoroughly describing the season, the sites, and included admissions, as well as by quantifying the heterogeneity of our estimates. In this way, we are able to visualise the relative impact of the different influenza strains on diverse risk factors, including age, comorbidity, pregnancy, and obesity [12]. Furthermore, we restricted our analysis to periods with influenza circulation [27], took into account risk by calendar date [28], as well as the clustering effect of site [10] by adjusting and modelling and, finally, compared PCR-detected influenza-positive admissions with influenza-negative admissions. We consider this a reasonable approach for describing the effect of influenza in individuals according to their risk profile [29]. In addition, to reduce bias and to allow us to describe the severe consequences of community-acquired influenza, we accepted only data from patients admitted within 7 days of onset of ILI symptoms and for whom swabbing was performed within 48 h of admission.

Even with a large dataset as the one accrued annually by the GIHSN sites, small numbers are a limitation. Splitting the data by strain and risk group can decrease group sizes, so that sufficient power is available only for detecting large differences (i.e., OR ≥2). This limitation can be only dealt with by increasing the number of participating sites and by pooling data across influenza seasons. In fact, the GIHSN continues to grow, and data pooling across seasons is underway.

Most hospital studies rely on the criteria of the physician providing care for influenza confirmation and employ historical database searching [15, 17, 18, 30–32]. This combined with different case definitions and laboratory methods can complicate comparisons between sites and seasons and between different studies. Our approach of using active surveillance, a shared core protocol, and PCR confirmation of influenza avoids these limitations. This approach has very recently begun to be employed by others and for other respiratory viruses [33].

## Conclusions

This report describes the results from the GIHSN during the 2014–2015 influenza season that were presented at the 2015 GIHSN Annual Meeting. During the 2014–2015 influenza season, the network included 27 hospitals in six countries (Russian Federation, Czech Republic, Turkey, China, Spain, and Brazil). This offered us the opportunity to describe the characteristics of severe disease related to influenza by time, person, and strain and to describe IVE across a wide geographical area in the Northern Hemisphere.

We found that influenza is associated with severe outcomes during an extended period in the Northern Hemisphere and that comorbidity and pregnancy were significant risk factors for severe influenza illness. The distribution and impact of the three influenza virus types (A(H1N1)pdm09, A(H3N2), and B) were similar to others’ reports. An important finding was that approximately 60 % of influenza-related hospital admissions were in healthy subjects with no known comorbidity.

Our results support the current WHO recommendations on the use of influenza vaccine [4], although for the 2014–2015, IVE was low due to a significant mismatch between the circulating and vaccine viruses. We also found that IVE was affected by age and the circulating strain. These findings highlight the need to develop vaccines that are more effective and cover a broader spectrum of influenza viruses.

## Abbreviations

AOR, adjusted odds ratio; CI, confidence interval; GIHSN, Global Influenza Hospital Surveillance Network; IVE, influenza vaccine effectiveness; OR, odds ratio; RT-PCR, reverse transcription-polymerase chain reaction

## Additional files

Additional file 1: Table S1. Characteristics of participating hospitals during the 2014–2015 season. (PDF 101 kb) Additional file 2: Table S2. Diagnoses and presenting complaints used to identify admissions possibly related with an influenza infection. (PDF 86 kb) Additional file 3: Table S3. Protocol application across sites. (PDF 85 kb) Additional file 4: Table S4. Types of vaccines available at each site. (PDF 69 kb) Additional file 5: Table S5. Site heterogeneity in the risk of a positive influenza result in included admissions. (PDF 11 kb) Additional file 6: Figure S1. Heterogeneity between sites in the OR of admission with a positive influenza result. (PDF 33 kb) Additional file 7: Figure S2. aOR and number of admissions with influenza by age group and virus strain. (PDF 86 kb) Additional file 8: Figure S3. aOR and number of admissions with influenza by virus strain in patients with one or more comorbidity compared to patients without comorbidity. (PDF 77 kb) Additional file 9: Figure S4. aOR and number of admissions with influenza by chronic underlying comorbidity and virus strain. (PDF 52 kb) Additional file 10: Table S6. Predicted probability of admission with influenza for women 15 to 45 years of age. (PDF 8 kb) Additional file 11: Figure S5. Probability of severe outcome by RT-PCR result. (PDF 83 kb) Additional file 12: Figure S6. aOR by vaccination the current year (2014–2015) and the two previous years (2013–2014 and 2012–2013). (PDF 31 kb) Additional file 13: Figure S7. Site-specific IVE against all influenza types for all ages. (PDF 43 kb) Additional file 14: Table S7. Sensitivity analysis. (PDF 106 kb) Additional file 15: Figure S8. Statistical methods to account for data clustering by site. (PDF 31 kb)
