# Supplementary material for: Influenza epidemiology and influenza vaccine effectiveness during the 2014–2015 season: annual report from the Global Influenza Hospital Surveillance Network
Source: BMC Public Health. 2016 Aug 22;16(Suppl 1):757. doi: 10.1186/s12889-016-3378-1 (PMC5001209; doi:10.1186/s12889-016-3378-1)
Supplement: Additional file 1: Table S1. — Characteristics of participating hospitals during the 2014–2015 season. (PDF 101 kb) [file 12889_2016_3378_MOESM1_ESM.pdf]

Table S1. Characteristics of participating hospitals during the 2014–2015 season

Cities (Country)	Hospital	Type of hospital	Total no. of beds	No. of beds monitored during study[a]	Type of wards monitored during study
St. Petersburg (Russian Federation)	City Infectious Diseases Hospital #30	Infectious disease for adults	300	300	Adults (≥17 y)
	Children's Infectious Diseases Hospital #5	Multi-type hospital for children	650	0	Infectious disease (0–16 y)
	Children's City Hospital #4	Multi-type hospital for children	370	0	Infectious disease (0–16 y)
Moscow (Russian Federation)	Emergency Respiratory Infection City Hospital #1	Viral infectious disease	806	506	Acute respiratory disease, paediatric, pregnant, ICU
Prague (Czech Republic)	Hospital na Bulovce, Prague	General hospital (former University hospital)	1007	506	Department of Infectious Disease respiratory diseases ward
Istanbul, Ankara, Bursa (Turkey	Hacettepe Univ. Adult Hospital	University	561	116	Acute medicine, emergency medicine
	Gazi Univ. Hospital	University	1068	229	Adult emergency, infectious disease
	Istanbul Faculty of Medicine	University	1353		Paediatrics: ICU, infectious, emergency, allergy
	Uludağ University Paediatric Hospital	University paediatric	108	0	All medical wards, ICU
	Dr. Lutfi Kirdar Kartal Research Hospital	General, Training and research	750	180	All medical wards, ICU
Beijing (China)	Changping District Hospital	General	576	241	Respiratory medicine, paediatrics, ICU
	The First Hospital of Huairou	General	651	298	Respiratory medicine, paediatrics, ICU
	Daxing District Hospital	General	918	459	Respiratory medicine, paediatrics, ICU
	Miyun County Hospital	General	541	276	Respiratory medicine, paediatrics, ICU
Valencia (Spain)	General de Castellón	General	580	451	All medical wards, ICU
	La Plana	General	201	100	All medical wards, ICU
	Arnau de Vilanova	General	308	190	All medical wards, ICU
	La Fe	General	1013	316	All medical wards, ICU
	Doctor Peset	General	540	211	All medical wards, ICU
	La Ribera	General	301	110	All medical wards, ICU
	San Juan	General	350	230	All medical wards, ICU
	Elda	General	514	410	All medical wards, ICU
	General Alicante	General	758	376	All medical wards, ICU
	Vinalopó	General	194	90	All medical wards, ICU
Rio de Janeiro (Brazil)	Hospital Albert Sabin	General (public)	267	126	Emergency, ICU, general pediatric, cardiology and pneumonia
	Hospital Quinta D'Or	General (private)	350	Not available	General and pediatric ward, clinical and pediatric ICU
	Hospital de Clínicas	University	310	Not available	ICU, semi-intensive care unit (adult), pediatric emergency service

[a] Medical beds excluding surgical beds
