# Supplementary material for: Influenza epidemiology and influenza vaccine effectiveness during the 2014–2015 season: annual report from the Global Influenza Hospital Surveillance Network
Source: BMC Public Health. 2016 Aug 22;16(Suppl 1):757. doi: 10.1186/s12889-016-3378-1 (PMC5001209; doi:10.1186/s12889-016-3378-1)
Supplement: Additional file 2: Table S2. — Diagnoses and presenting complaints used to identify admissions possibly related with an influenza infection. (PDF 86 kb) [file 12889_2016_3378_MOESM2_ESM.pdf]

Table S2. Diagnoses and presenting complaints used to identify admissions possibly related with an influenza infection

Patients ≥5 years of age	ICD-9 Codes	ICD-10 Codes
Acute respiratory infection	382.9; 460–466	J00–J06, J20–J22, H66.90
Acute myocardial infarction or acute coronary syndrome	410–411 and 413–414	I20–I25.9
Asthma	493–493.92	J45.2–J45.22, J45.9–J45.998, J44–J44.9
Heart failure	428–429.0	I50–I50.9; I51.4
Pneumonia and influenza	480–488	J09–J18
COPD	490, 491, 492, 496	J40–J44.9
Myalgia	729.1	M79.1
Metabolic failure (diabetic coma, renal dysfunction, acid-base disturbances, alterations to the water balance)	250.1–250.3; 584–586; 276–277	E11.9, E10.9, E11.65, E10.65, E10.11, E11.01, E10.641, E11.641, E10.69, E11.00, E10.10, E11.69, N17.0, N17.1, N17.2, N17.8, N17.9, N18.1, N18.2, N18.3, N18.4, N18.5, N18.6 N18.9, N19, E87.0, E87.1, E87.2, E87.3, E87.4, E87.5, E87.6, E87.70, E87.71, E87.79, E86.0, E86.1
Altered consciousness, convulsions, febrile convulsions	780.01–780.02; 780.09; 780.31–780.32	R40.20, R40.4, R40.0, R40.1, R56.00, R56.01
Dyspnoea/respiratory abnormality	786.0	R06.0, R06–R06.9
Respiratory abnormality	786.00	R06.9
Shortness of breath	786.05	R06.02
Respiratory abnormality nec	786.09	R06.3, R06.00, R06.09, R06.83
Respiratory symptoms/chest symptoms	786.9	R06.89
Fever or fever unknown origin or non-specified	780.6–780.60	R50, R50.9
Cough	786.2	R05
Sepsis, systemic inflammatory response syndrome	995.90–995.94	R65.10, R65.11, R65.20, A41.9
**Patients 0–4 years of age**	**ICD 9 Codes**	**ICD 10 Codes**
Acute upper or lower respiratory disease	382.9; 460–466	J00–J06, J20–J22
Dyspnoea, breathing anomaly, shortness of breath, tachypnea	786.0; 786.00; 786.05–786.07; 786.09; 786.9	R06.0, R06, R06.9, R06.3, R06.00, R06.09, R06.83, R06.02, R06.82, R06.2, R06.89
Asthma	493–493.92	J45.2–J45.22, J45.9–J45.998, J44–J44.9
Pneumonia and influenza	480 to 488	J09–J18
Heart failure	428–429.0	I50–I50.9; I51.4
Myalgia	729.1	M79.1
Altered consciousness, convulsions, febrile convulsions	780.01–780.02; 780.09; 780.31–780.32	R40.20, R40.4, R40.0, R40.1, R56.00, R56.01
Fever or fever unknown origin or non-specified	780.6–780.60	R50, R50.9
Cough	786.2	R05
Gastrointestinal manifestations	009.0; 009.3	A09.0; A09.9
Sepsis, systemic inflammatory response syndrome	995.90–995.94	R65.10, R65.11, R65.20, A41.9

Admissions possibly related to influenza infection were based on Hayden FG, de Jong MD: Human influenza: Pathogenesis, clinical features, and management; In Webster RG, Monto AS, Braciale TJ, Lamb RA, editors. Textbook of Influenza. Chichester, West Sussex, UK, Wiley Blackwell, 2013; 374–391.
