# Supplementary material for: Influenza epidemiology and influenza vaccine effectiveness during the 2014–2015 season: annual report from the Global Influenza Hospital Surveillance Network
Source: BMC Public Health. 2016 Aug 22;16(Suppl 1):757. doi: 10.1186/s12889-016-3378-1 (PMC5001209; doi:10.1186/s12889-016-3378-1)
Supplement: Additional file 3: Table S3. — Protocol application across sites. (PDF 85 kb) [file 12889_2016_3378_MOESM3_ESM.pdf]

Table S3. Protocol application across sites

Characteristic	St. Petersburg (Russian Federation)	Moscow (Russian Federation)	Prague (Czech Republic)	Istanbul, Ankara, Bursa (Turkey)	Beijing (China)	Valencia (Spain)	Rio de Janeiro (Brazil)
Screening diagnosis	Admission diagnosis: influenza, acute respiratory illness, severe acute respiratory infections, pneumonia, croup, and bronchitis	Admission diagnosis possibly associated with an influenza infection (Table S2)	Admission diagnosis possibly associated with an influenza infection (Table S2)	Admission diagnosis possibly associated with an influenza infection (Table S2)	Admission diagnosis possibly associated with an influenza infection (Table S2)	Admission diagnosis possibly associated with an influenza infection (Table S2)	Admission diagnosis possibly associated with an influenza infection (Table S2)
Residency definition	Resident of St. Petersburg ≥6 months	Resident of Moscow ≥6 months and has not been away for more 1 month	Permanent residence in Prague or Central Bohemian Region	Resident of the city where the hospital is located ≥6 months	Resident of the district of where the hospital is located ≥6 months	Pertaining to hospital catchment area and resident for ≥6 months	Resident of the region (Albert Sabin) or metropolitan area (Quinta d'Or) where the hospital is located; resident of the region where the hospital is located ≥6 months (Hospital de Clinicas)
Recruited by	Doctors	Doctors, nurses, head of wards	Doctors, nurses	Doctors, resident doctors	Doctors, nurses	Full-time trained nurses	Doctors, nurses
Study start criteria	Week with ≥5 laboratory-confirmed influenza cases	One or more influenza positive cases in 2 consecutive weeks	≥1 positive patient per week for 2 consecutive weeks (November)	2 consecutive weeks with ≥1 cases	Defined by the national surveillance system	2 consecutive weeks with ≥2 cases	Entire year (Albert Sabin), not available (Hospital de Clinicas), 2 consecutive weeks with ≥2 cases (Quinta d'Or)
Study end criteria	Week with no laboratory-confirmed influenza cases	Week with no laboratory-confirmed influenza cases	No influenza-positive patients during the previous 4 weeks or reaching of the maximum total number of patients per 2014–2015 study period	2 consecutive weeks with no cases	Defined by the national surveillance system	2 consecutive weeks with <3 cases	2 consecutive weeks with no cases (Quinta d'Or, Hospital de Clinicas), not available (Albert Sabin)
Actual study period (epidemiological weeks with flu cases ascertained)	2015 wk 4 – 2015 wk 22	2015 wk 2 – 2015 wk 17	2015 wk 2 – 2015 wk 16	2015 wk 2 – 2015 wk 24	2014 wk 40 – 2015 wk 17	2014 wk 50 – 2015 wk 12	2015 wk 1 to wk 52 (Albert Sabin), 2015 wk 10 to wk 39 (Quinta D'Or, Hospital de Clinicas)
