# Supplementary material for: Influenza epidemiology and influenza vaccine effectiveness during the 2014–2015 season: annual report from the Global Influenza Hospital Surveillance Network
Source: BMC Public Health. 2016 Aug 22;16(Suppl 1):757. doi: 10.1186/s12889-016-3378-1 (PMC5001209; doi:10.1186/s12889-016-3378-1)
Supplement: Additional file 4: Table S4. — Types of vaccines available at each site. (PDF 69 kb) [file 12889_2016_3378_MOESM4_ESM.pdf]

Table S4.Types of vaccines available at each site

Site	Vaccines available in 2014–2015
St. Petersburg, Russian Federation	Grippol “NPO Petrovax Farm” and “NPO Microgen”
	Grippol – plus “NPO Petrovax Farm”
	Vaxigrip
	Begrivac
	Fluarix
	Influvac
Moscow, Russian Federation	Grippol and Grippol plus
	Influvir
	Grippovac
	Influvac
Prague, Czech Republic	Vaxigrip
	Influvac
Istanbul, Turkey	Vaxigrip
	Fluarix
Beijing, China	Trivalent inactivated vaccines (mainly split virion and subunit)
Valencia, Spain	Vaxigrip
	Chriromas
	Optaflu
