# Supplementary material for: Influenza epidemiology and influenza vaccine effectiveness during the 2014–2015 season: annual report from the Global Influenza Hospital Surveillance Network
Source: BMC Public Health. 2016 Aug 22;16(Suppl 1):757. doi: 10.1186/s12889-016-3378-1 (PMC5001209; doi:10.1186/s12889-016-3378-1)
Supplement: Additional file 5: Table S5. — Site heterogeneity in the risk of a positive influenza result in included admissions. (PDF 11 kb) [file 12889_2016_3378_MOESM5_ESM.pdf]

Table S5. Site heterogeneity in the risk of a positive influenza result in included admissions

Recruiting site	Influenza-positive admissions	All admissions	% influenza positive	Crude OR	95% CI	Adjusted OR[a]	95% CI
All sites	N=2177	N=9164	26.1	.	.		.
St. Petersburg	602	2715	22.2	1.00	.	1.00	. .
Czech Republic	59	79	74.7	10.35	6.12-17.53	4.14	2.42-7.10
Turkey	71	614	11.6	0.46	0.35-0.60	0.29	0.22-0.39
Beijing	273	1214	24.3	1.10	0.93-1.29	0.97	0.80-1.17
Valencia	737	3657	20.2	0.89	0.78-1.00	0.50	0.41-0.60

OR, odds ratio. CI, confidence interval.

[a]Site effect adjusted by sex, age, comorbidity, hospitalization during the previous year, time to swab, and calendar time, with St. Petersburg as a reference
