# Supplementary figures and images for: Influenza epidemiology and influenza vaccine effectiveness during the 2014–2015 season: annual report from the Global Influenza Hospital Surveillance Network
Source: BMC Public Health. 2016 Aug 22;16(Suppl 1):757. doi: 10.1186/s12889-016-3378-1 (PMC5001209; doi:10.1186/s12889-016-3378-1)

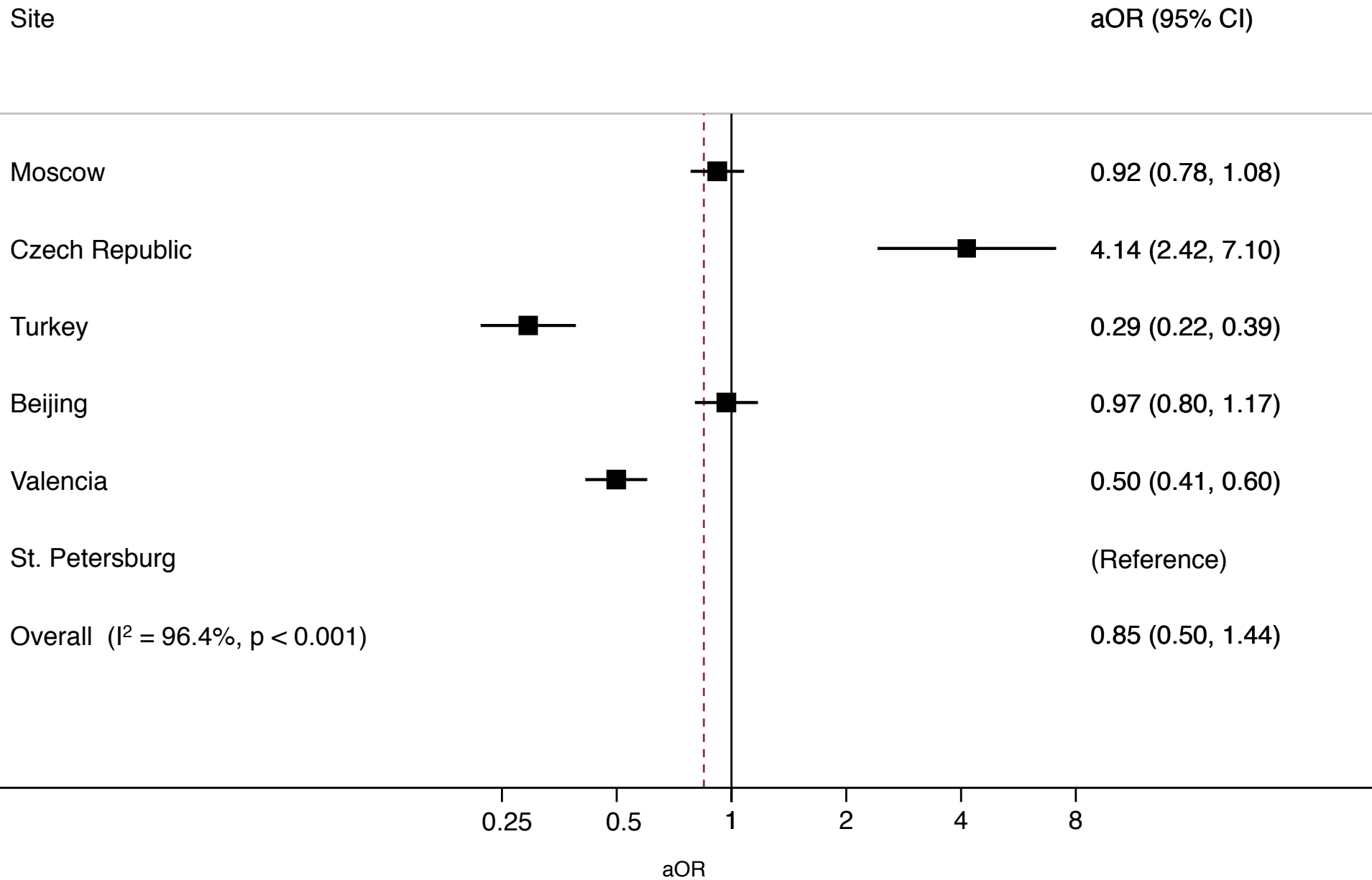

Supplement: Additional file 6: Figure S1. — Heterogeneity between sites in the OR of admission with a positive influenza result. (PDF 33 kb) [file 12889_2016_3378_MOESM6_ESM.pdf]

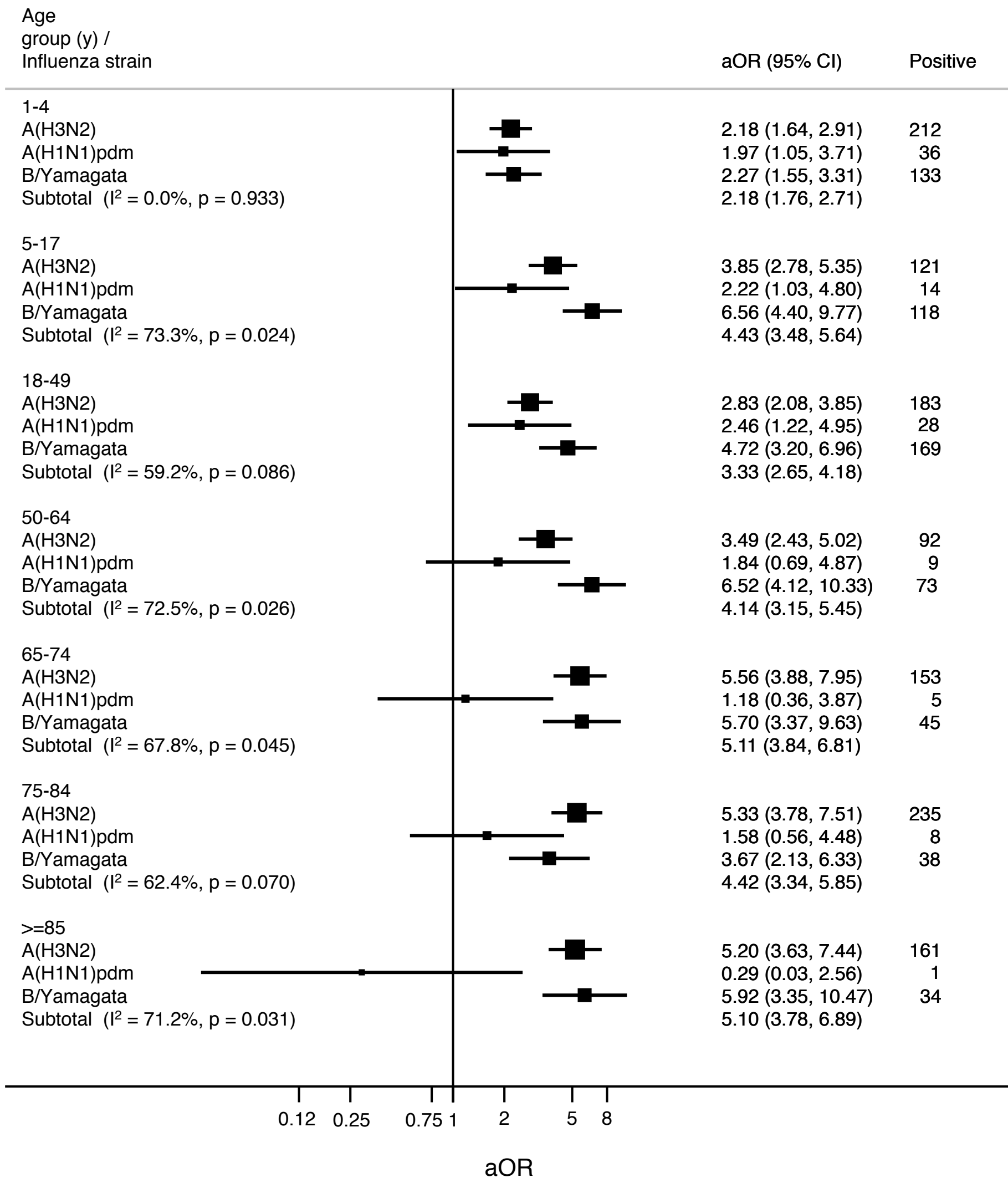

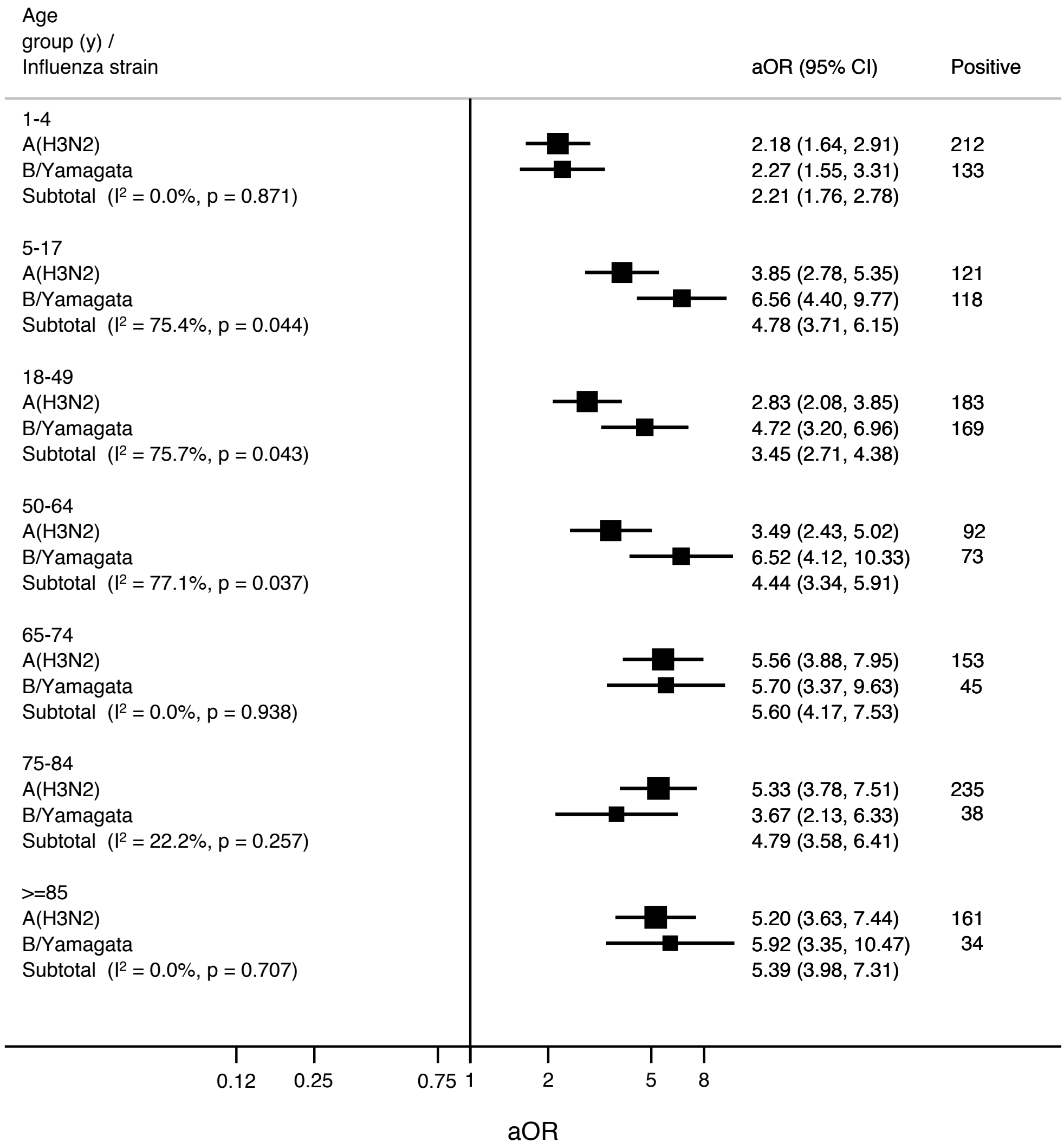

Supplement: Additional file 7: Figure S2. — aOR and number of admissions with influenza by age group and virus strain. (PDF 86 kb) [file 12889_2016_3378_MOESM7_ESM.pdf]

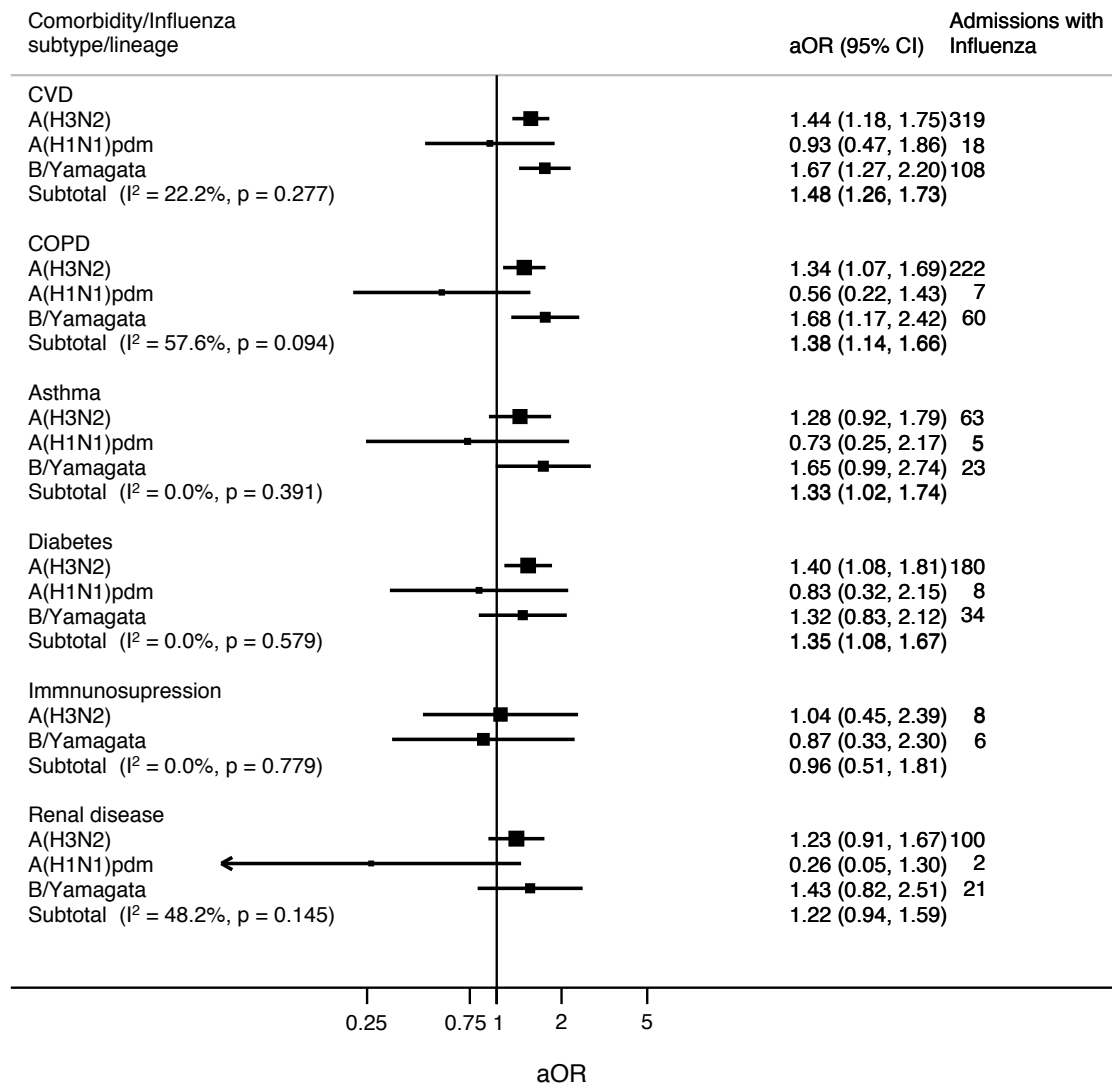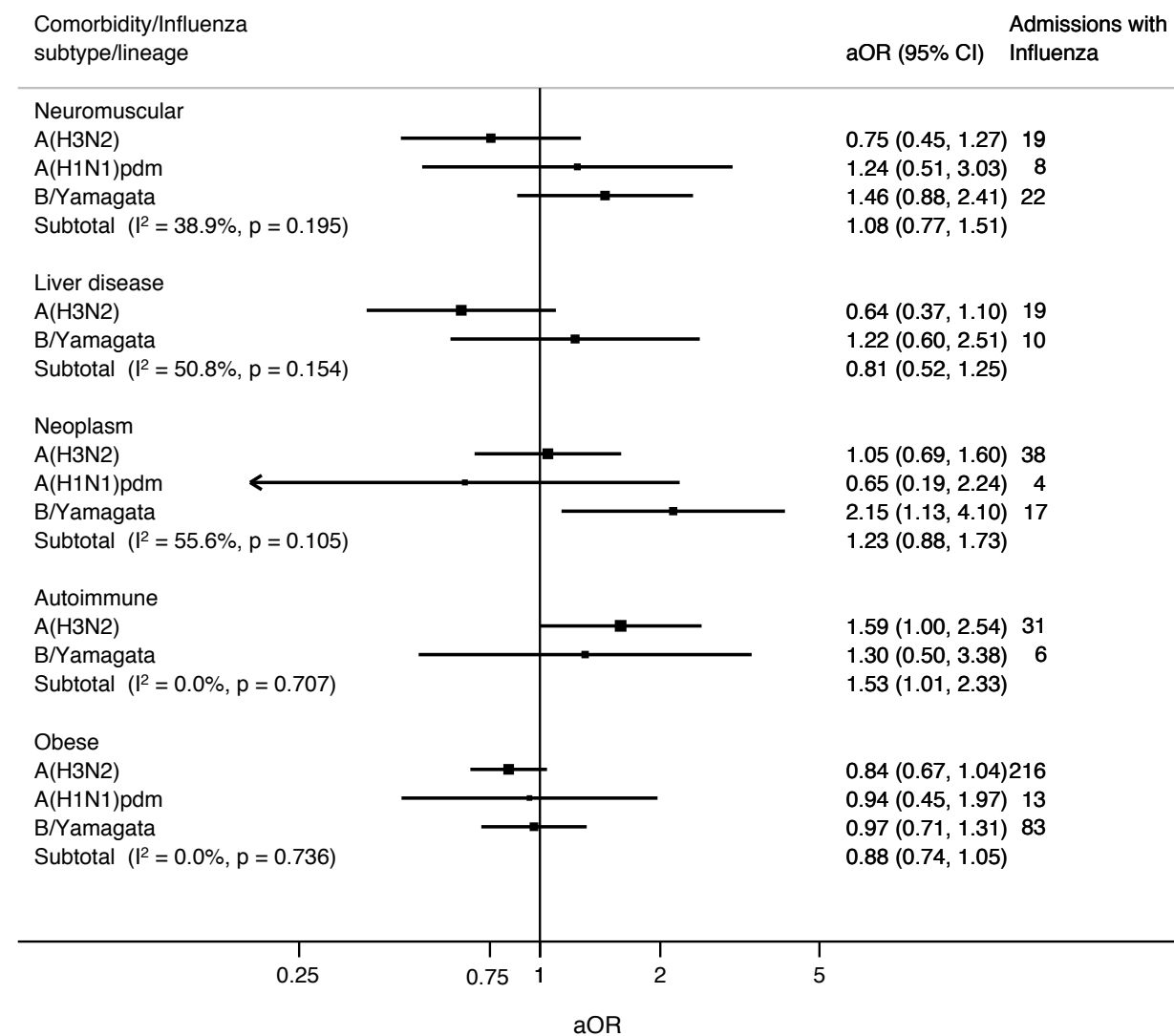

Supplement: Additional file 9: Figure S4. — aOR and number of admissions with influenza by chronic underlying comorbidity and virus strain. (PDF 52 kb) [file 12889_2016_3378_MOESM9_ESM.pdf]

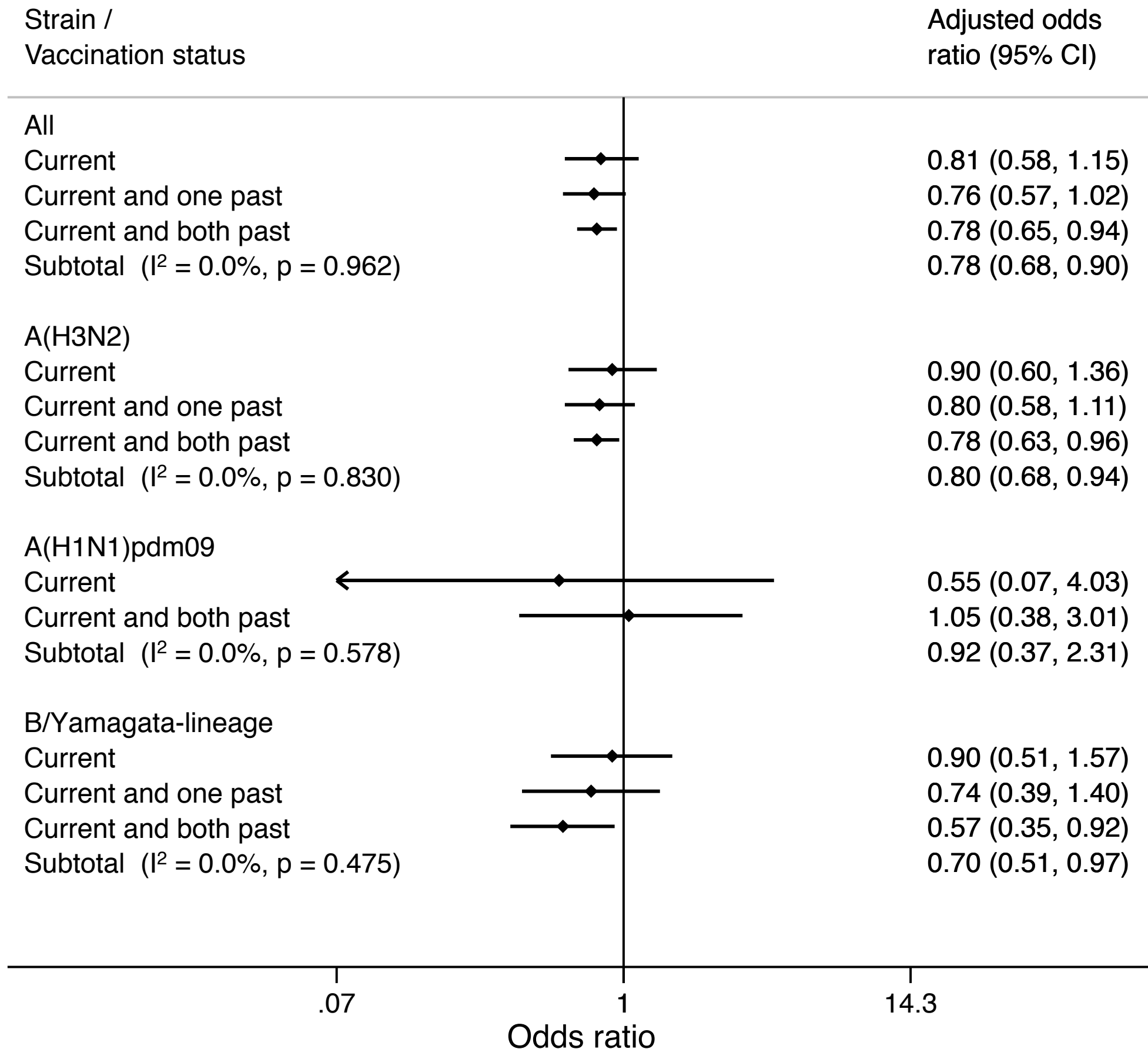

Supplement: Additional file 12: Figure S6. — aOR by vaccination the current year (2014–2015) and the two previous years (2013–2014 and 2012–2013). (PDF 31 kb) [file 12889_2016_3378_MOESM12_ESM.pdf]

Analysis / Site

IVE (95% CI)

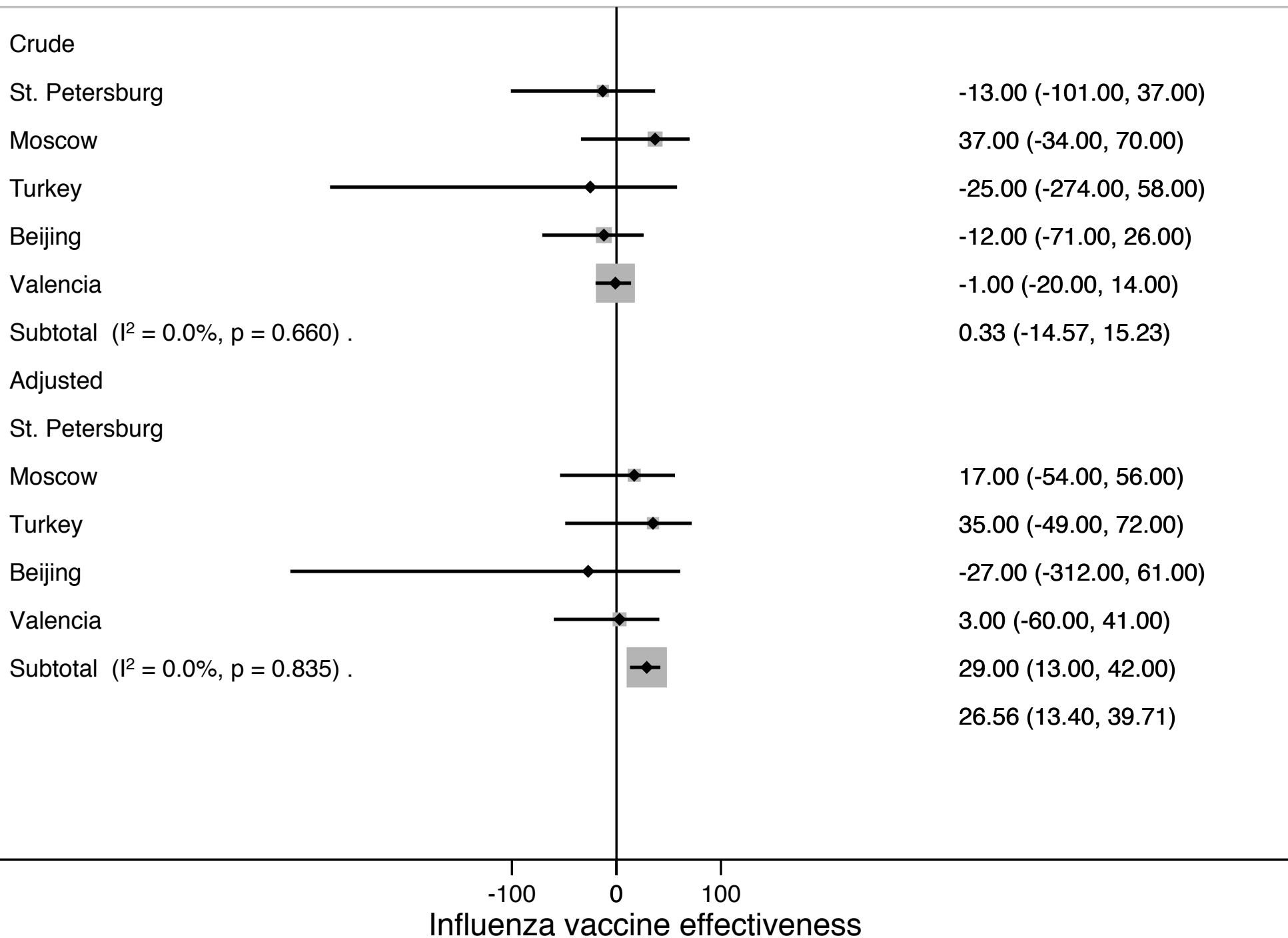

Supplement: Additional file 13: Figure S7. — Site-specific IVE against all influenza types for all ages. (PDF 43 kb) [file 12889_2016_3378_MOESM13_ESM.pdf]

Statistical methods to  
account for data clustering by site

IVE (95% CI)

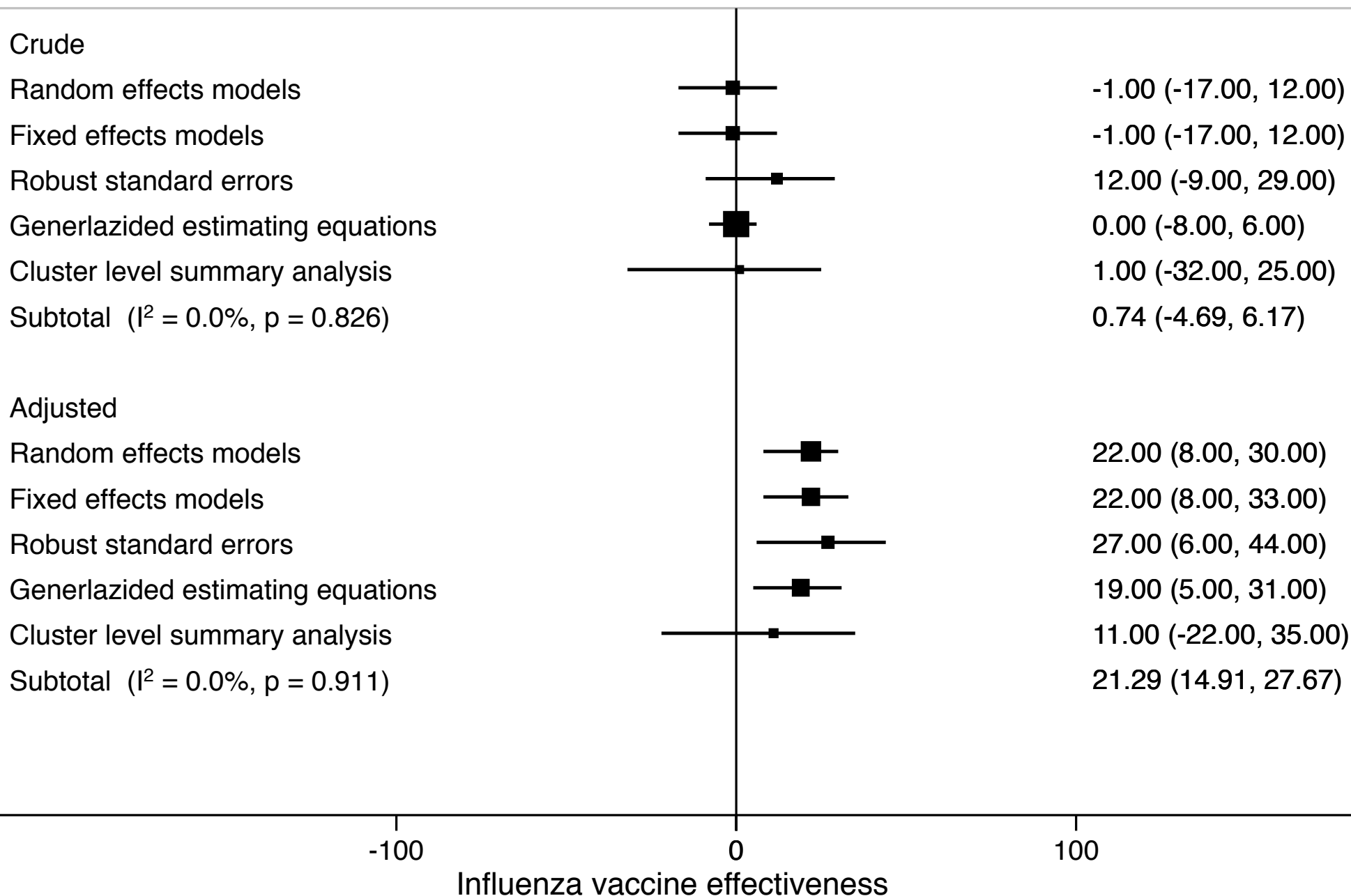

Supplement: Additional file 15: Figure S8. — Statistical methods to account for data clustering by site. (PDF 31 kb) [file 12889_2016_3378_MOESM15_ESM.pdf]
