# Supplementary material for: Influenza epidemiology and influenza vaccine effectiveness during the 2014–2015 season: annual report from the Global Influenza Hospital Surveillance Network
Source: BMC Public Health. 2016 Aug 22;16(Suppl 1):757. doi: 10.1186/s12889-016-3378-1 (PMC5001209; doi:10.1186/s12889-016-3378-1)
Supplement: Additional file 8: Figure S3. — aOR and number of admissions with influenza by virus strain in patients with one or more comorbidity compared to patients without comorbidity. (PDF 77 kb) [file 12889_2016_3378_MOESM8_ESM.pdf]

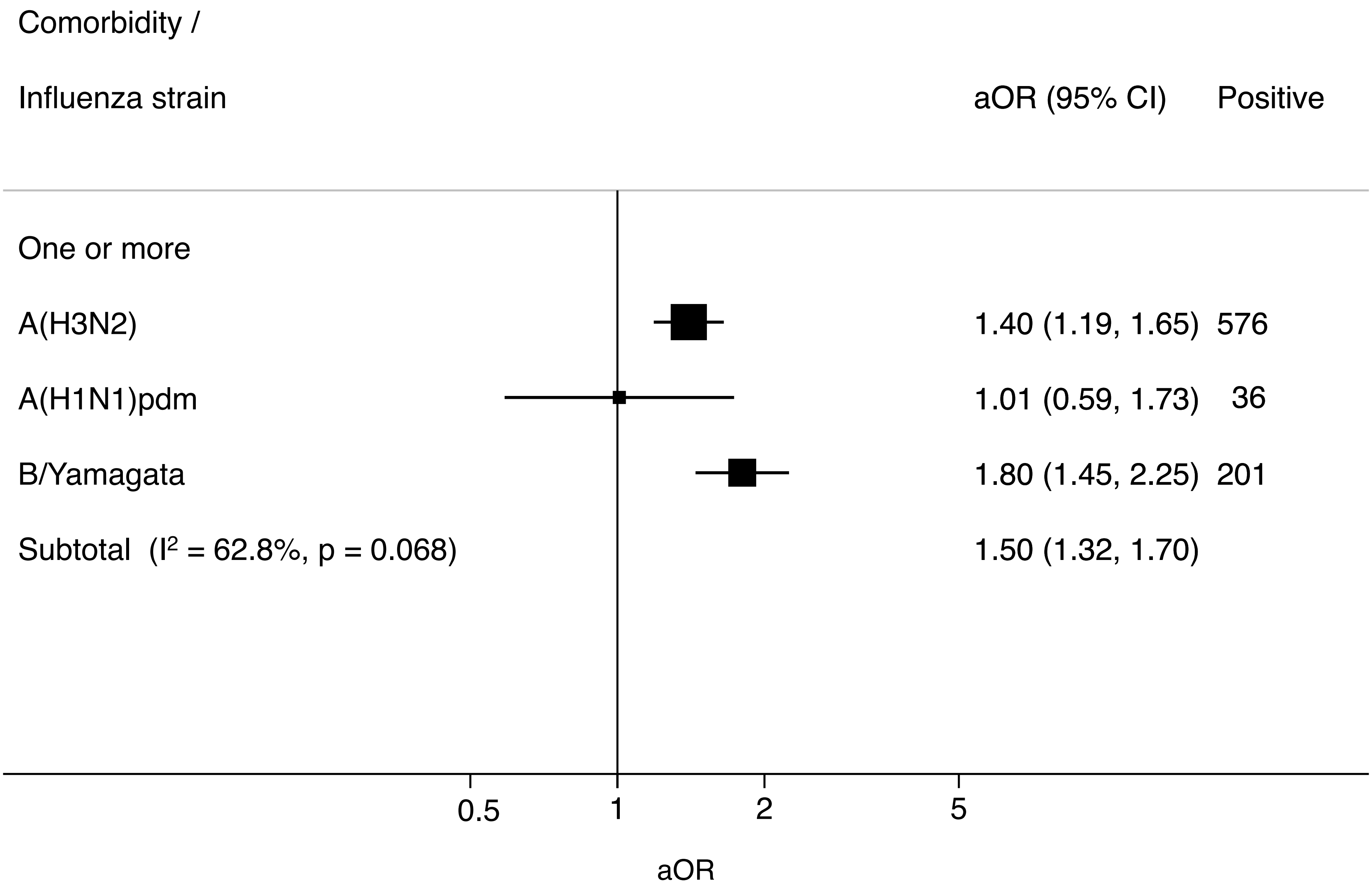

Comorbidity /
Influenza strain
aOR (95% CI)
Positive
One or more
A(H3N2)
1.40 (1.19, 1.65)
576
A(H1N1)pdm
1.01 (0.59, 1.73)
36
B/Yamagata
1.80 (1.45, 2.25)
201
Subtotal (I2 = 62.8%, p = 0.068)
1.50 (1.32, 1.70)
0.5
1
2
5
aOR

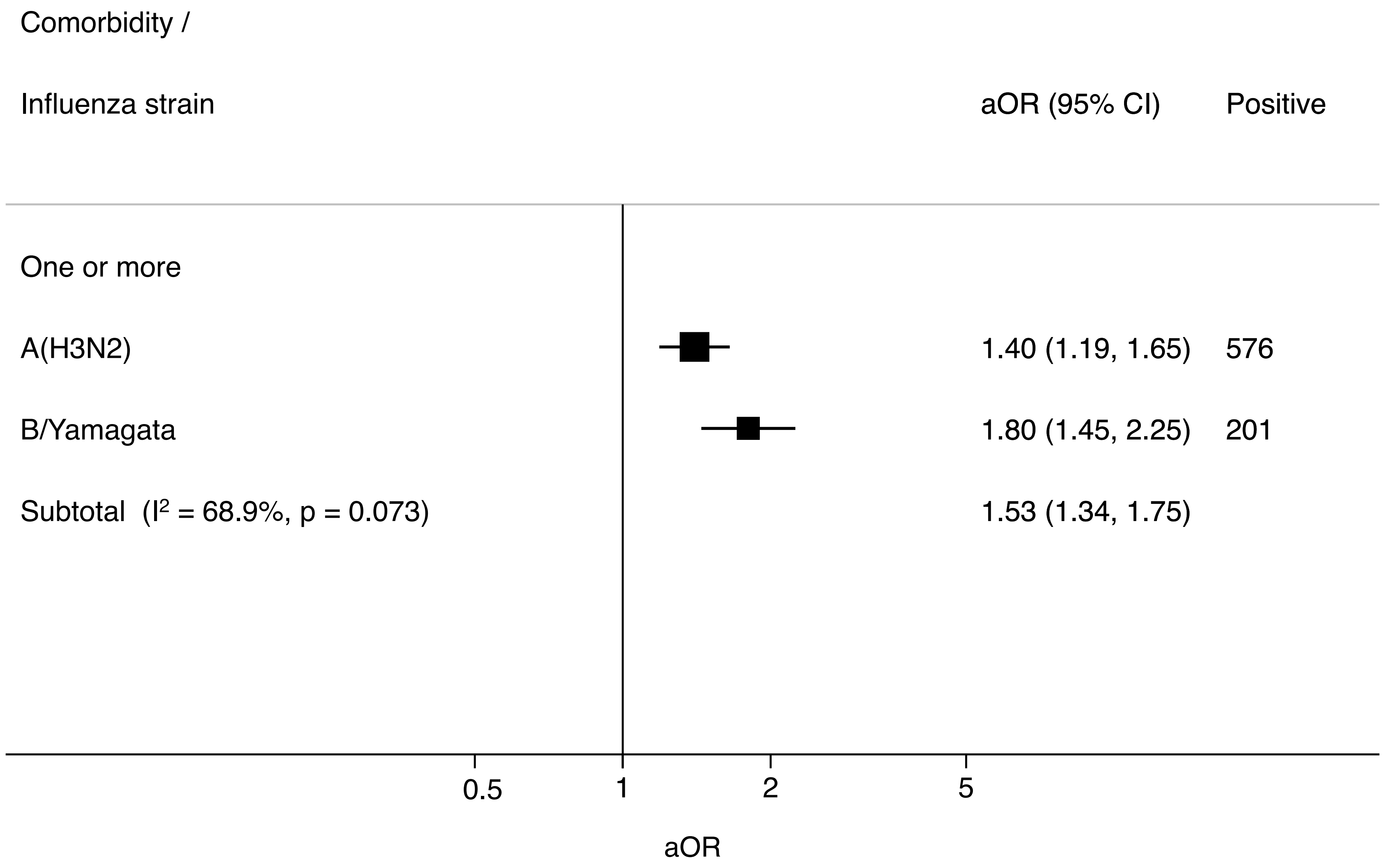
Comorbidity /
Influenza strain
aOR (95% CI)
Positive
One or more
A(H3N2)
1.40 (1.19, 1.65)
576
B/Yamagata
1.80 (1.45, 2.25)
201
Subtotal (I^2 = 68.9%, p = 0.073)
1.53 (1.34, 1.75)
0.5
1
2
5
aOR
