# Supplementary material for: Influenza epidemiology and influenza vaccine effectiveness during the 2014–2015 season: annual report from the Global Influenza Hospital Surveillance Network
Source: BMC Public Health. 2016 Aug 22;16(Suppl 1):757. doi: 10.1186/s12889-016-3378-1 (PMC5001209; doi:10.1186/s12889-016-3378-1)
Supplement: Additional file 10: Table S6. — Predicted probability of admission with influenza for women 15 to 45 years of age. (PDF 8 kb) [file 12889_2016_3378_MOESM10_ESM.pdf]

Table S6. Predicted probability of admission with influenza for women 15 to 45 years of age

Pregnancy status	Comorbidity	Probability (%)[a]	95% CI
Not pregnant	No	31.6	25.2-38.1
	Yes	32.9	23.0-42.7
First trimester	No	38.6	37.6-39.5
	Yes	80.5	79.0-81.9
Second trimester	No	44.1	40.9-47.4
	Yes	56.0	50.6-61.5
Third trimester	No	43.0	39.4-46.5
	Yes	55.2	48.0-62.5

[a]Adjusted by social class, smoking habits, time to swab, calendar time, and the cluster effect of recruiting site
