# Supplementary material for: Influenza epidemiology and influenza vaccine effectiveness during the 2014–2015 season: annual report from the Global Influenza Hospital Surveillance Network
Source: BMC Public Health. 2016 Aug 22;16(Suppl 1):757. doi: 10.1186/s12889-016-3378-1 (PMC5001209; doi:10.1186/s12889-016-3378-1)
Supplement: Additional file 11: Figure S5. — Probability of severe outcome by RT-PCR result. (PDF 83 kb) [file 12889_2016_3378_MOESM11_ESM.pdf]

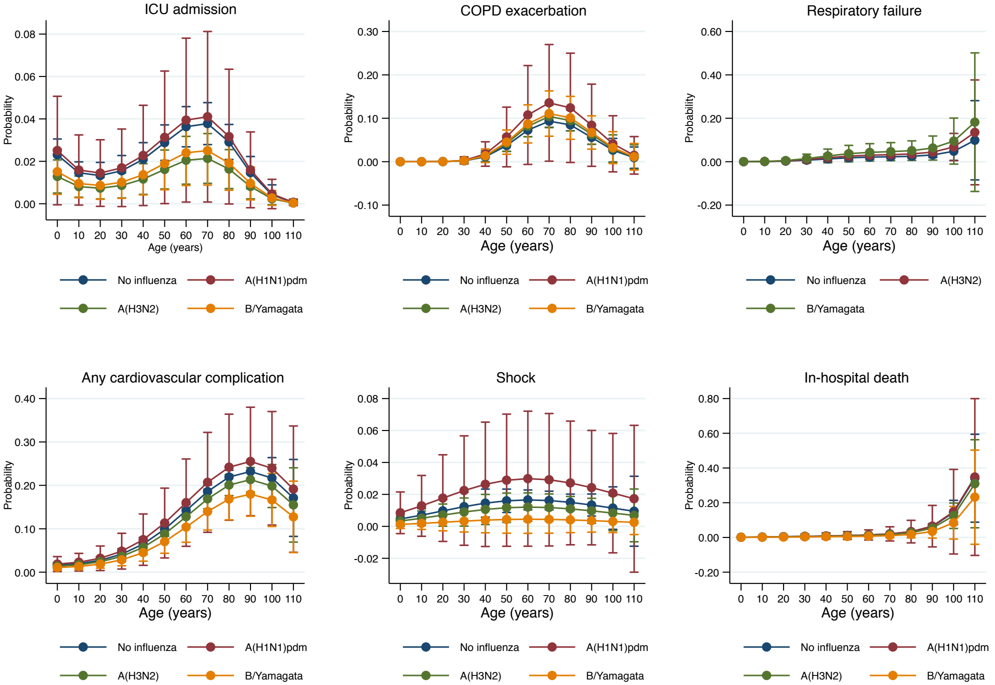
ICU admission
COPD exacerbation
Respiratory failure
Any cardiovascular complication
Shock
In-hospital death
Probability
Age (years)
0 10 20 30 40 50 60 70 80 90 100 110
No influenza
A(H1N1)pdm
A(H3N2)
B/Yamagata
