# Supplementary material for: Influenza epidemiology and influenza vaccine effectiveness during the 2014–2015 season: annual report from the Global Influenza Hospital Surveillance Network
Source: BMC Public Health. 2016 Aug 22;16(Suppl 1):757. doi: 10.1186/s12889-016-3378-1 (PMC5001209; doi:10.1186/s12889-016-3378-1)
Supplement: Additional file 14: Table S7. — Sensitivity analysis. (PDF 106 kb) [file 12889_2016_3378_MOESM14_ESM.pdf]

Table S7. Sensitivity analysis

			All			Vaccine target group		
**Sensitivity analysis**	**Strain**	**Age group**	**Adjusted IVE**	**95% CI**	**P interaction**	**Adjusted IVE**	**95% CI**	**P interaction**
Exclusion of pregnant women	Any	All ages	-2	-18 to 12		21	8 to 33	
	H3N2	All ages	-7	-26 to 10		20	4 to 33	
	H1N1	All ages	22	-94 to 68		24	-92 to 70	
	B/Yamagata	All ages	13	-21 to 38		31	2 to 51	
	Any							
	Any	<65 y	-7	-41 to 18	0.0589	-5	-38 to 21	0.0583
	H3N2	≥65 y	21	6 to 34		24	8 to 37	
	H3N2	<65 y	-19	-64 to 14	0.0246	-17	-65 to 17	0.031
	H1N1	≥65 y	24	7 to 38		25	7 to 39	
	H1N1	<65 y	9	-196 to 72	0.9258	8	-201 to 72	0.7638
	B/Yamagata	≥65 y	47	-128 to 88		58	-81 to 90	
	B/Yamagata	<65 y	-1	-63 to 37	0.1577	2	-60 to 40	0.2634
		≥65 y	37	1 to 59		33	-7 to 58	
Excluding vaccinated <14 d from illness onset	Any	All ages	-1	-17 to 13		21	8 to 33	
	H3N2	All ages	-5	-24 to 11		20	4 to 33	
	H1N1	All ages	25	-84 to 70		27	-82 to 71	
	B/Yamagata	All ages	16	-17 to 40		31	2 to 52	
	Any	<65 y	-4	-36 to 20	0.091	-5	-38 to 21	0.0568
	Any	≥65 y	21	5 to 34		24	9 to 37	
	H3N2	<65 y	-15	-58 to 17	0.037	-16	-63 to 17	0.033
	H3N2	≥65 y	24	7 to 38		25	7 to 39	
	H1N1	<65 y	18	-167 to 75	0.998	15	-176 to 74	0.9906
	H1N1	≥65 y	18	-210 to 78		32	-158 to 82	
	B/Yamagata	<65 y	7	-50 to 42	0.257	3	-58 to 40	0.2724
	B/Yamagata	≥65 y	36	0 to 59		33	-7 to 58	
Vaccinated according to registration records only	Any	All ages	-6	-22 to 9		18	4 to 30	
	H3N2	All ages	-7	-26 to 9		19	2 to 32	
	H1N1	All ages	7	-131 to 63		-	-	
	B/Yamagata	All ages	2	-37 to 30		25	-12 to 45	
	Any	<65 y	-17	-56 to 12	0.0365	-16	-55 to 14	0.0262
	Any	≥65 y	18	2 to 31		22	6 to 35	
	H3N2	<65 y	-25	-77 to 11	0.0195	-28	-84 to 11	0.0157
	H3N2	≥65 y	22	5 to 36		24	7 to 38	
	H1N1	<65 y	-11	-265 to 66	0.8523	-	-	
	H1N1	≥65 y	5	-253 to 75		-	-	
	B/Yamagata	<65 y	-16	-90 to 29	0.1739	-16	-91 to 30	0.2121
	B/Yamagata	≥65 y	27	-14 to 53		25	-19 to 52	

Abbreviations: CI, confidence interval; IVE, influenza vaccine effectiveness
